# Supplementary figures and images for: Nutrient availability is a dominant predictor of soil bacterial and fungal community composition after nitrogen addition in subtropical acidic forests
Source: PLoS One. 2021 Feb 23;16(2):e0246263. doi: 10.1371/journal.pone.0246263 (PMC7901772; doi:10.1371/journal.pone.0246263)

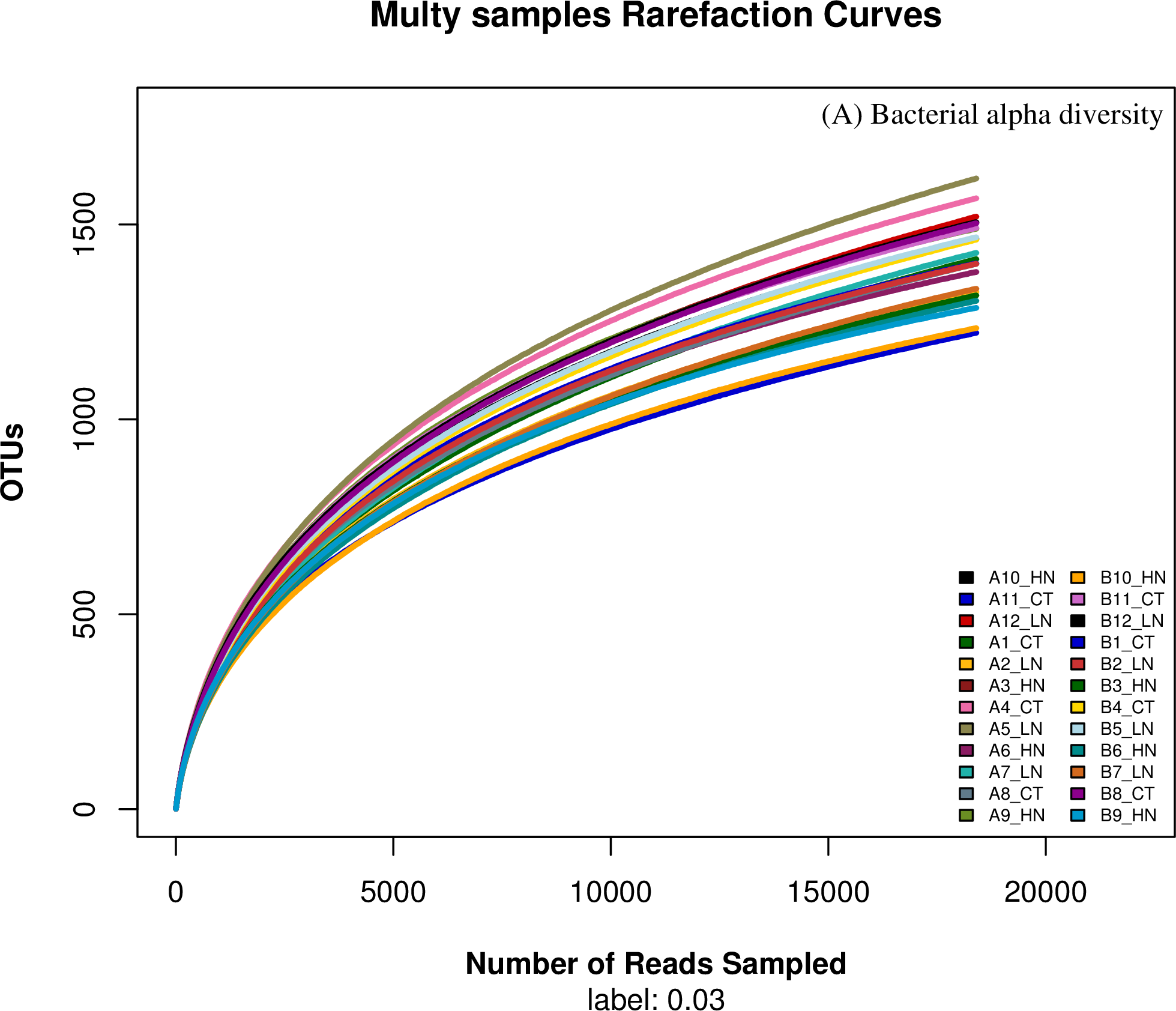

Supplement: S1 Fig — Rarefaction curves of bacterial (A) and fungal (B) α-diversity. The amount of sequencing data is deemed to be reasonable when the curves are flat and more data will produce only a small number of new species. (ZIP) [file pone.0246263.s001.zip › S1A_Fig.tif]

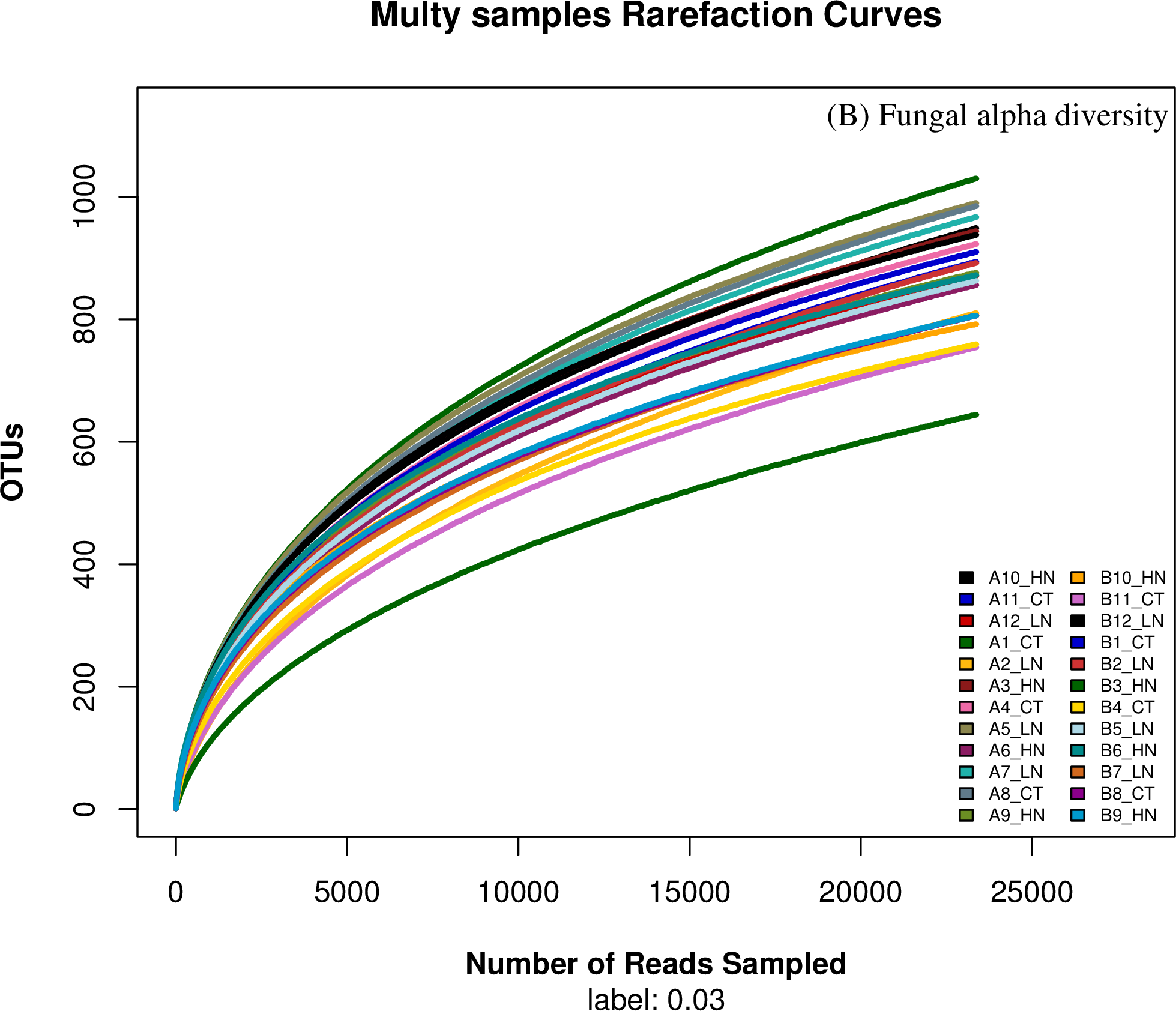

Supplement: S1 Fig — Rarefaction curves of bacterial (A) and fungal (B) α-diversity. The amount of sequencing data is deemed to be reasonable when the curves are flat and more data will produce only a small number of new species. (ZIP) [file pone.0246263.s001.zip › S1B_Fig.tif]

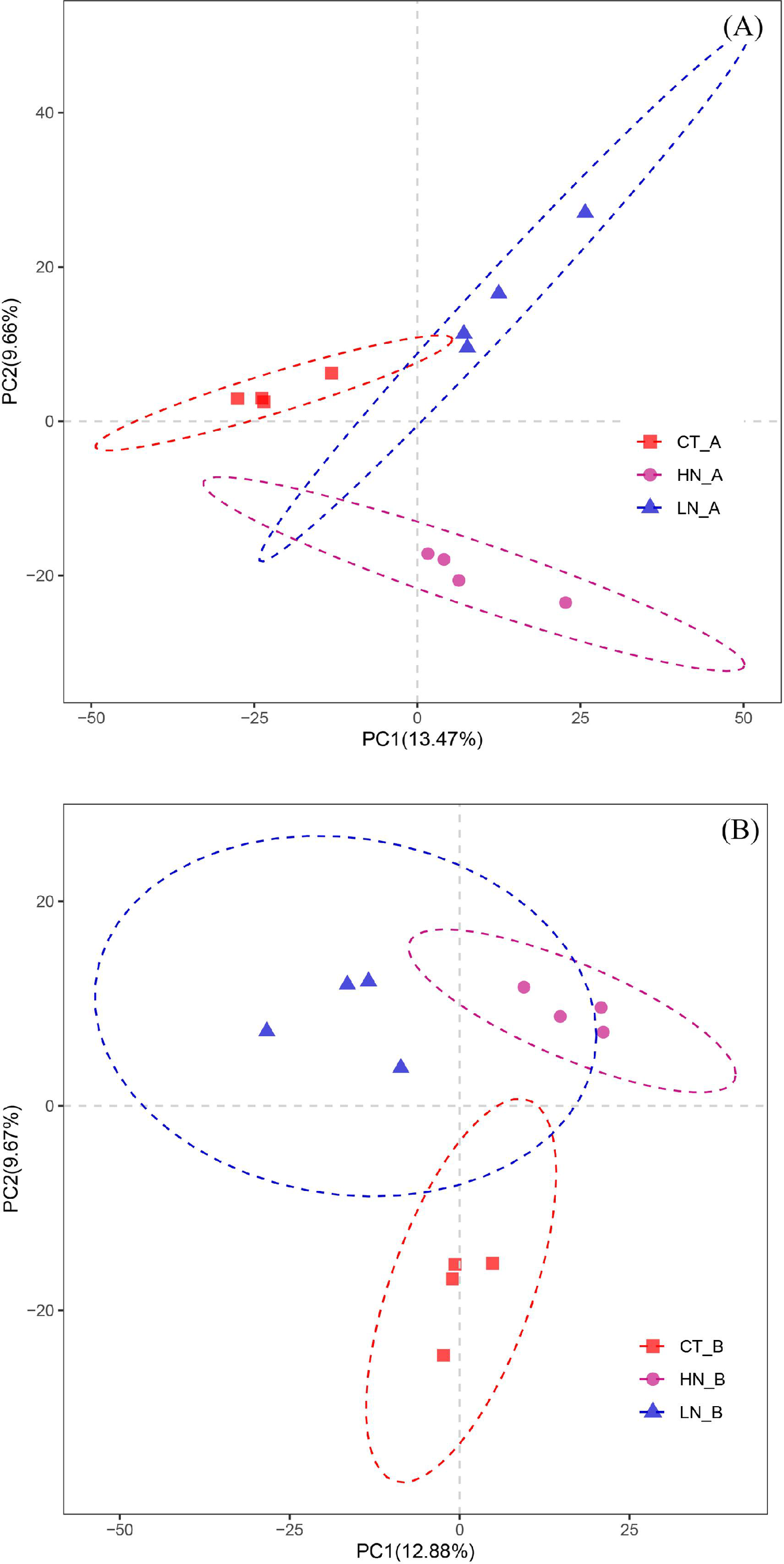

Supplement: S2 Fig — Partial Least Squares Discrimination Analysis (PLS-DA) of the soil bacterial (A, B) and fungal (C, D) community structure in the topsoil and subsoil under different N addition treatments. The variation in community composition was determined based on the abundance of OTUs. CT, LN, and HN, represent the control, low, and high nitrogen addition treatments, respectively. (ZIP) [file pone.0246263.s002.zip › S2A,B_Fig.tif]

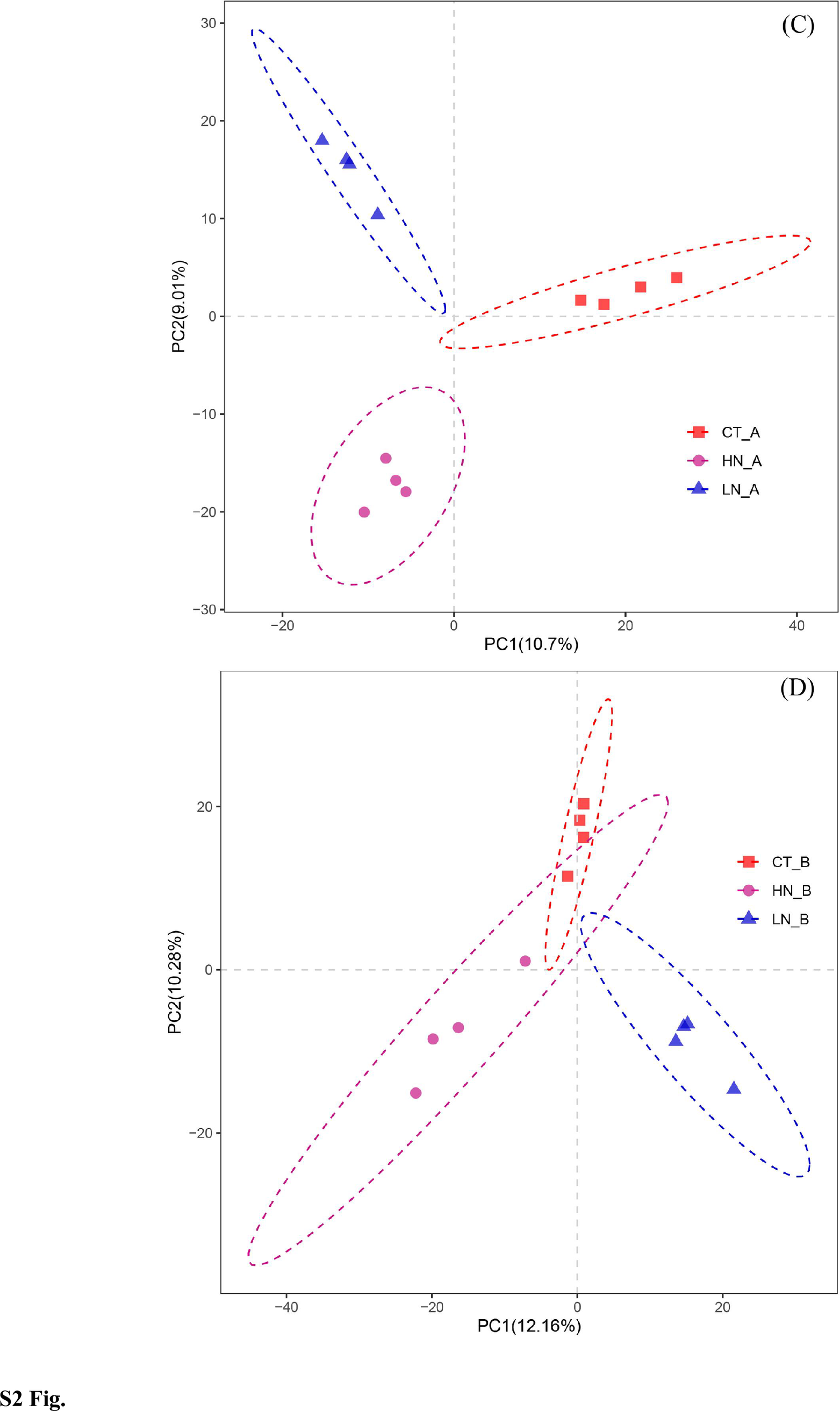

Supplement: S2 Fig — Partial Least Squares Discrimination Analysis (PLS-DA) of the soil bacterial (A, B) and fungal (C, D) community structure in the topsoil and subsoil under different N addition treatments. The variation in community composition was determined based on the abundance of OTUs. CT, LN, and HN, represent the control, low, and high nitrogen addition treatments, respectively. (ZIP) [file pone.0246263.s002.zip › S2C,D_Fig.tif]

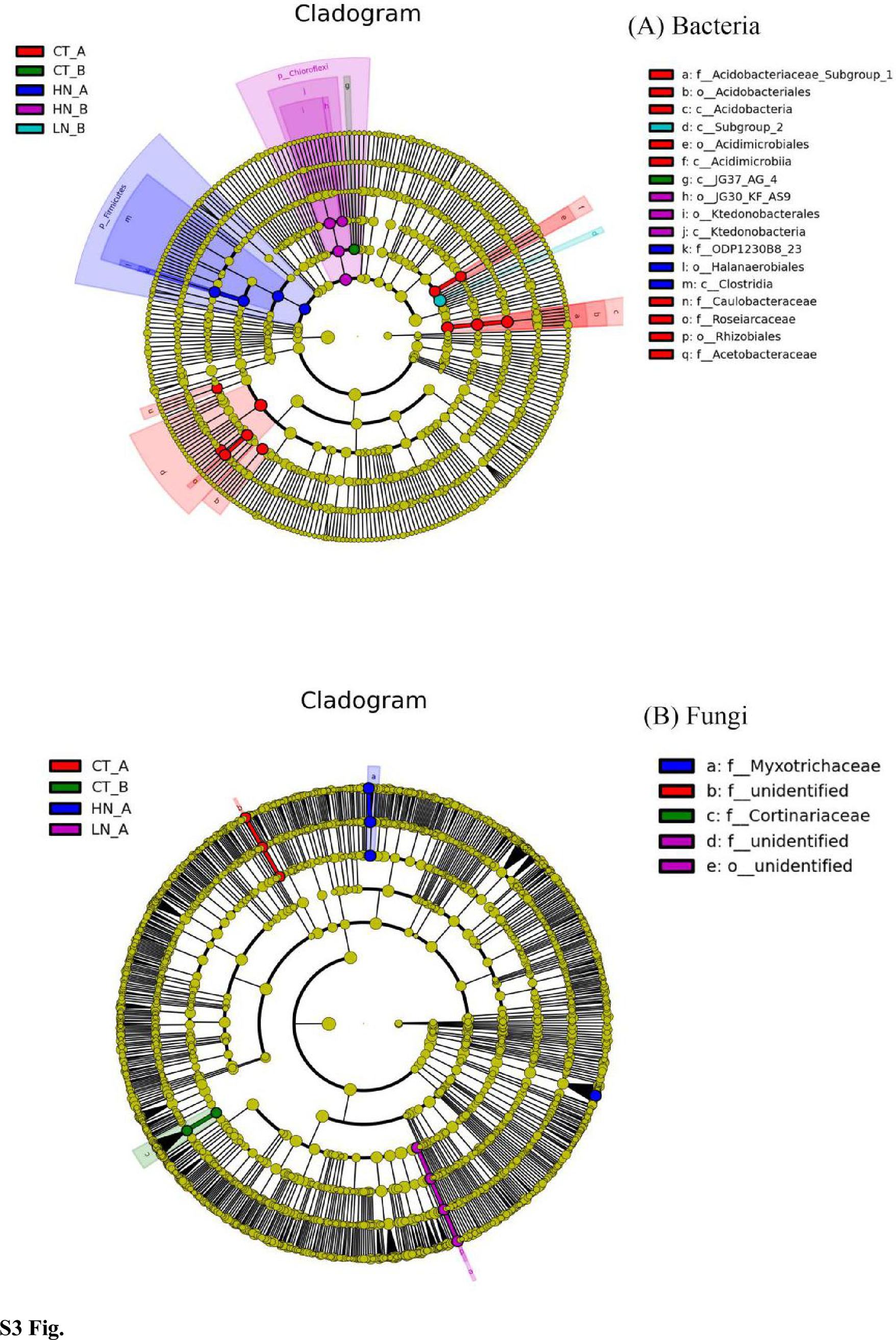

Supplement: S3 Fig — Cladograms depicting bacterial (A) and fungal (B) species variation under different N addition treatments. LDA analysis was performed to obtain these cladograms. (TIF) [file pone.0246263.s003.tif]
