# Supplementary material for: Nutrient availability is a dominant predictor of soil bacterial and fungal community composition after nitrogen addition in subtropical acidic forests
Source: PLoS One. 2021 Feb 23;16(2):e0246263. doi: 10.1371/journal.pone.0246263 (PMC7901772; doi:10.1371/journal.pone.0246263)
Supplement: S1 Table — (DOCX) [file pone.0246263.s004.docx]

**S1 Table. Primer sequences used in this study.**

| Primer Name | Primer sequence (5ʹ-3ʹ) |
| --- | --- |
| 16S v3-v4 forward primer | GTACTCCTACGGGAGGCAGCA |
| 16S v3-v4 reverse primer | GTGGACTACHVGGGTWTCTAAT |
| ITS1 forward primer | CTTGGTCATTTAGAGGAAGTAA |
| ITS1 reverse primer | TGCGTTCTTCATCGATGC |
