# Supplementary material for: Nutrient availability is a dominant predictor of soil bacterial and fungal community composition after nitrogen addition in subtropical acidic forests
Source: PLoS One. 2021 Feb 23;16(2):e0246263. doi: 10.1371/journal.pone.0246263 (PMC7901772; doi:10.1371/journal.pone.0246263)
Supplement: S2 Table — (DOCX) [file pone.0246263.s005.docx]

**S2 Table. Effects of different N-addition treatments at different soil depths based on 16S rDNA gene copy numbers, ITS gene copy numbers and fungi-to-bacteria ratios.**

|  | 16S | ITS | F:B |
| --- | --- | --- | --- |
| CT-A | 1.20E+07 | 1.63E+06 | 0.250 |
| LN-A | 1.86E+08 | 3.80E+06 | 0.347 |
| HN-A | 1.39E+05 | 3.12E+05 | 0.801 |
| CT-B | 4.01E+08 | 3.41E+06 | 0.013 |
| LN-B | 1.05E+09 | 1.38E+06 | 0.002 |
| HN-B | 3.26E+07 | 2.87E+05 | 0.042 |

**CT, control; LN, low nitrogen addition; HN, high nitrogen addition; A, topsoil; B, subsoil.**
