# Supplementary material for: Nutrient availability is a dominant predictor of soil bacterial and fungal community composition after nitrogen addition in subtropical acidic forests
Source: PLoS One. 2021 Feb 23;16(2):e0246263. doi: 10.1371/journal.pone.0246263 (PMC7901772; doi:10.1371/journal.pone.0246263)
Supplement: S3 Table — (DOCX) [file pone.0246263.s006.docx]

**S3 Table. Pearson's correlations of microbial abundance to soil properties and microbial biomass.**

| Soil properties | Bacterial abundance | Fungal abundance | Fungi-to-Bacteria ratio |
| --- | --- | --- | --- |
| pH（H_2_^O）^ | 0.385 | 0.164 | -0.607** |
| pH (KCl) | 0.776** | -0.037 | -0.159 |
| SMC | -0.38 | 0.231 | 0.440* |
| CEC | -0.305 | 0.466* | 0.094 |
| NH_4_^+-N^ | -.493* | 0.057 | 0.353 |
| NO_3_^--N^ | -0.174 | -0.29 | 0.508* |
| available_N | -0.459* | 0.033 | 0.387 |
| DOC | -0.32 | 0.101 | 0.391 |
| SOC | -0.443* | 0.163 | 0.498* |
| TN | -0.464* | 0.222 | 0.458* |
| TP | -0.481* | 0.133 | 0.333 |
| CN Ratio | 0.015 | -0.179 | 0.3 |
| MBC | -0.232 | 0.122 | 0.467* |
| MBN | -0.402 | 0.222 | 0.584** |

Note: SMC, soil moisture content; CEC, soil cation exchange capacity; NH4+-N, ammonium N; NO3−-N, nitrate N; DOC, dissolved organic carbon; SOC, soil organic carbon; TN, total N; TP, total P.

*p < 0.05, **p < 0.01.
