# Supplementary material for: Nutrient availability is a dominant predictor of soil bacterial and fungal community composition after nitrogen addition in subtropical acidic forests
Source: PLoS One. 2021 Feb 23;16(2):e0246263. doi: 10.1371/journal.pone.0246263 (PMC7901772; doi:10.1371/journal.pone.0246263)
Supplement: S4 Table — (DOCX) [file pone.0246263.s007.docx]

**S4 Table. Good's coverage values of each group.**

|  | Index | Bacterial goods_coverage | Fungal  goods_coverage |
| --- | --- | --- | --- |
| Topsoil | CT | 97.29% | 98.43% |
|  | LN | 97.13% | 98.50% |
|  | HN | 97.44% | 98.56% |
|  | p value | 0.26 | 0.269 |
| Subsoil | CT | 97.30% | 98.67% |
|  | LN | 97.40% | 98.60% |
|  | HN | 97.60% | 98.75% |
|  | p value | 0.03 | 0.066 |

**CT, control; LN, low nitrogen addition; HN, high nitrogen addition**
