# Supplementary material for: Nutrient availability is a dominant predictor of soil bacterial and fungal community composition after nitrogen addition in subtropical acidic forests
Source: PLoS One. 2021 Feb 23;16(2):e0246263. doi: 10.1371/journal.pone.0246263 (PMC7901772; doi:10.1371/journal.pone.0246263)
Supplement: S5 Table — (DOCX) [file pone.0246263.s008.docx]

**S5 Table. Effects of N addition on soil bacterial and fungal α-diversity indices.**

| Diversity indices | Topsoil (0-10 cm) | | | | Subsoil (10-20 cm) | | | |
| --- | --- | --- | --- | --- | --- | --- | --- | --- |
|  | CT | LN | HN | p | CT | LN | HN | p |
| B_chao1 | 1982.88 | 2100.99 | 1939.25 | 0.43 | 2010.63a | 1925.25ab | 1782.60b | 0.05* |
| B_goods_coverage | 0.97 | 0.97 | 0.97 | 0.26 | 0.97b | 0.97b | 0.98a | 0.03* |
| B_observed_species | 1399.70 | 1472.98 | 1458.68 | 0.64 | 1463.68a | 1426.68a | 1285.48b | 0.00** |
| B_PD_whole_tree | 98.94 | 104.14 | 104.37 | 0.42 | 106.97a | 104.65a | 96.04b | 0.00** |
| B_shannon | 8.32 | 8.30 | 8.46 | 0.33 | 8.31a | 8.23a | 7.99b | 0.03* |
| B_simpson | 0.99 | 0.99 | 0.99 | 0.17 | 0.99 | 0.99 | 0.99 | 0.22 |
| F_chao1 | 1412.96 | 1373.87 | 1363.66 | 0.69 | 1192.84 | 1293.34 | 1165.80 | 0.12 |
| F_goods_coverage | 0.98 | 0.98 | 0.99 | 0.27 | 0.99ab | 0.98b | 0.99a | 0.07 |
| F_observed_species | 958.00 | 909.43 | 906.23 | 0.49 | 807.23 | 874.68 | 778.45 | 0.24 |
| F_PD_whole_tree | 171.36 | 164.95 | 162.81 | 0.52 | 146.12 | 159.80 | 146.23 | 0.17 |
| F_shannon | 6.28 | 5.87 | 6.29 | 0.63 | 5.42 | 6.13 | 5.40 | 0.69 |
| F_simpson | 0.96 | 0.93 | 0.95 | 0.67 | 0.89 | 0.95 | 0.82 | 0.61 |
