# Supplementary material for: Nutrient availability is a dominant predictor of soil bacterial and fungal community composition after nitrogen addition in subtropical acidic forests
Source: PLoS One. 2021 Feb 23;16(2):e0246263. doi: 10.1371/journal.pone.0246263 (PMC7901772; doi:10.1371/journal.pone.0246263)
Supplement: Attachment — Submitted filename: PONE-D-20-10592_reviewer.pdf [file pone.0246263.s009.pdf]

PLOS ONE

Nutrient availability is a dominant predictor of soil bacterial and fungal community composition after nitrogen addition in subtropic al acidic forest

--Manuscript Draft--

**Manuscript Number:**	PONE-D-20-10592
**Article Type:**	Research Article
**Full Title:**	Nutrient availability is a dominant predictor of soil bacterial and fungal community composition after nitrogen addition in subtropic al acidic forest
**Short Title:**	Nutrient availability is a predictor of soil microbial community composition after nitrogen addition in forest
**Corresponding Author:**	Yuehmin Chen<br>Fujian Normal University<br>Fuzhou, CHINA
**Keywords:**	Available N; DOC; Nitrogen addition; soil microbial community
**Abstract:**	Nutrient (N) addition to forest ecosystems significantly influences underground microbial diversity, community structure, and ecosystem functioning. Among them, nitrogen addition is notable in China, especially in the southeast of China. However, our knowledge about the influence of N addition to underground soil microbial diversity and community in subtropical forests is still incomplete. We randomly chose 12 experimental plots in Pinus taiwanensis forest of the Daiyun mountain Nature Reserve located in Fujian Province to perform simulated N addition treatments, determine the soil microbial community response, and identify the dominant factor determining microbial community composition. This study included three N treatments, i.e., control (CT), low N addition (40 kg N ha^{-1} yr^{-1}), high N addition (80 kg N ha^{-1} yr^{-1}) and two depths, 0−10 cm (topsoil) and 10−20 cm (subsoil), sampled in the growing season (May) of 2019. We investigated microbial diversity and community in the topsoil and subsoil using high-throughput sequencing of bacterial 16S rDNA genes and ITS fungal sequences. Results indicated that 1) soil DOC and available N (NH_4^+) were significantly decreased after nitrogen addition, 2) bacterial α-diversity in subsoil was significantly increased with N additions, and 3) soil nutrient availability, rather than pH, was a key environmental parameter determining the composition of the soil microbe community. Moreover, N addition affected both bacterial diversity and community composition more than those of fungi in subtropical forests.
**Order of Authors:**	Juyan Cui
	Xiaochun Yuan
	Qiufang Zhang
	Jiacong Zhou
	Kaimiao Lin
	Jianguo Xu
	Yaozhong Zeng
	Yue Wu
	Lei Cheng
	Quanxin Zeng
	Kongcan Mei
	Yuehmin Chen
**Additional Information:**	
**Question**	**Response**
**Financial Disclosure**	The research was supported by the National Natural Science Foundation of China (No.

Powered by Editorial Manager® and ProduXion Manager® from Aries Systems Corporation

Enter a financial disclosure statement that describes the sources of funding for the work included in this submission. Review the submission guidelines for detailed requirements. View published research articles from *PLOS ONE* for specific examples.<br><br>This statement is required for submission and **will appear in the published article** if the submission is accepted. Please make sure it is accurate.<br><br>**Unfunded studies**<br>Enter: *The author(s) received no specific funding for this work.*<br><br>**Funded studies**<br>Enter a statement with the following details:<br>• Initials of the authors who received each award<br>• Grant numbers awarded to each author<br>• The full name of each funder<br>• URL of each funder website<br>• Did the sponsors or funders play any role in the study design, data collection and analysis, decision to publish, or preparation of the manuscript?<br>• **NO** - Include this sentence at the end of your statement: *The funders had no role in study design, data collection and analysis, decision to publish, or preparation of the manuscript.*<br>• **YES** - Specify the role(s) played.<br><br>* typeset	31670620).
**Competing Interests**<br><br>Use the instructions below to enter a competing interest statement for this submission. On behalf of all authors, disclose any competing interests that could be perceived to bias this work—acknowledging all financial support and any other relevant financial or non-financial competing interests.<br><br>This statement **will appear in the published article** if the submission is accepted. Please make sure it is accurate. View published research articles	The authors have declared that no competing interests exist.

from *PLOS ONE* for specific examples.<br><br>**NO authors have competing interests**<br><br>Enter: *The authors have declared that no competing interests exist.*<br><br>**Authors with competing interests**<br><br>Enter competing interest details beginning with this statement:<br><br>*I have read the journal's policy and the authors of this manuscript have the following competing interests: [insert competing interests here]*<br><br>* typeset	
**Ethics Statement**<br><br>Enter an ethics statement for this submission. This statement is required if the study involved:<br><br>• Human participants<br>• Human specimens or tissue<br>• Vertebrate animals or cephalopods<br>• Vertebrate embryos or tissues<br>• Field research<br><br>Write "N/A" if the submission does not require an ethics statement.<br><br>General guidance is provided below. Consult the submission guidelines for detailed instructions. **Make sure that all information entered here is included in the Methods section of the manuscript.**	N/A

**Format for specific study types**<br><br>**Human Subject Research (involving human participants and/or tissue)**<br>• Give the name of the institutional review board or ethics committee that approved the study<br>• Include the approval number and/or a statement indicating approval of this research<br>• Indicate the form of consent obtained (written/oral) or the reason that consent was not obtained (e.g. the data were analyzed anonymously)<br><br>**Animal Research (involving vertebrate animals, embryos or tissues)**<br>• Provide the name of the Institutional Animal Care and Use Committee (IACUC) or other relevant ethics board that reviewed the study protocol, and indicate whether they approved this research or granted a formal waiver of ethical approval<br>• Include an approval number if one was obtained<br>• If the study involved *non-human primates*, add *additional details* about animal welfare and steps taken to ameliorate suffering<br>• If anesthesia, euthanasia, or any kind of animal sacrifice is part of the study, include briefly which substances and/or methods were applied<br><br>**Field Research**<br><br>Include the following details if this study involves the collection of plant, animal, or other materials from a natural setting:<br>• Field permit number<br>• Name of the institution or relevant body that granted permission	
**Data Availability**<br><br>Authors are required to make all data underlying the findings described fully available, without restriction, and from the time of publication. PLOS allows rare exceptions to address legal and ethical concerns. See the PLOS Data Policy and FAQ for detailed information.	Yes - all data are fully available without restriction

Powered by Editorial Manager® and ProduXion Manager® from Aries Systems Corporation

A Data Availability Statement describing where the data can be found is required at submission. Your answers to this question constitute the Data Availability Statement and **will be published in the article**, if accepted.<br><br>**Important:** Stating 'data available on request from the author' is not sufficient. If your data are only available upon request, select 'No' for the first question and explain your exceptional situation in the text box.<br><br>Do the authors confirm that all data underlying the findings described in their manuscript are fully available without restriction?	
**Describe where the data may be found in full sentences. If you are copying our sample text, replace any instances of XXX with the appropriate details.**<br><br>• If the data are **held or will be held in a public repository**, include URLs, accession numbers or DOIs. If this information will only be available after acceptance, indicate this by ticking the box below. For example: *All XXX files are available from the XXX database (accession number(s) XXX, XXX.).*<br>• If the data are all contained **within the manuscript and/or Supporting Information files**, enter the following: *All relevant data are within the manuscript and its Supporting Information files.*<br>• If neither of these applies but you are able to provide **details of access elsewhere**, with or without limitations, please do so. For example:<br><br>*Data cannot be shared publicly because of [XXX]. Data are available from the XXX Institutional Data Access / Ethics Committee (contact via XXX) for researchers who meet the criteria for access to confidential data.*<br><br>*The data underlying the results presented in the study are available from (include the name of the third party*	All relevant data are within the manuscript and its Supporting Information files.

and contact information or URL).<br>• This text is appropriate if the data are owned by a third party and authors do not have permission to share the data.<br><br>* typeset	
Additional data availability information:	Tick here if the URLs/accession numbers/DOIs will be available only after acceptance of the manuscript for publication so that we can ensure their inclusion before publication.

Powered by Editorial Manager® and ProduXion Manager® from Aries Systems Corporation

manuscript Click here to download Manuscript plos one1st.docx

Nutrient availability is a dominant predictor of soil bacterial and fungal community composition after nitrogen addition in subtropical acidic forest

Juyan Cui[1,2,3], Xiaochun Yuan [1,2,4], Qiufang Zhang [1,2], Jiacong Zhou [1,2], Kaimiao Lin[4], Jianguo Xu[5], Yaozhong Zeng[1,2], Yue Wu[1,2], Lei Cheng [1,2], Quanxin Zeng [1,2], Kongcan Mei [1,2], Yuehmin Chen[1,2,6]*

Affiliation:

1 College of Geographical Science, Fujian Normal University, Fuzhou 350007, China

2 State Key Laboratory of Subtropical Mountain Ecology, Fujian Normal University, Fuzhou 350007, China

3 Architectural Engineering Institute, Tongling University, Tongling 244000, China

4 Tourism College, Wuyi University, Wuyishan 354300, China

5 Daiyun Mountain National Nature Reserve Administration Bureau, Quanzhou 362500, China

6 Institute of Geography, Fujian Normal University, Fuzhou 350007, China

***Corresponding authors:**

Yuehmin Chen

E-mail: ymchen@fjnu.edu.cn;

Telephone: +86-591-83465013

Fax: +86-591-83465397

Abstract

Nutrient (N) addition to forest ecosystems significantly influences underground microbial diversity, community structure, and ecosystem functioning. Among them, nitrogen addition is notable in China, especially in the southeast of China. However, our knowledge about the influence of N addition to underground soil microbial diversity and community in subtropical forests is still incomplete. We randomly chose 12 experimental plots in *Pinus taiwanensis* forest of the Daiyun mountain Nature Reserve located in Fujian Province to perform simulated N addition treatments, determine the soil microbial community response, and identify the dominant factor determining microbial community composition. This study included three N treatments, i.e., control (CT), low N addition (40 kg N ha^{-1} yr^{-1}), high N addition (80 kg N ha^{-1} yr^{-1}) and two depths, 0−10 cm (topsoil) and 10−20 cm (subsoil), sampled in the growing season (May) of 2019. We investigated microbial diversity and community in the topsoil and subsoil using high-throughput sequencing of bacterial 16S rDNA genes and ITS fungal sequences. Results indicated that 1) soil DOC and available N (NH_4^+) were significantly decreased after nitrogen addition, 2) bacterial α-diversity in subsoil was significantly increased with N additions, and 3) soil nutrient availability, rather than pH, was a key environmental parameter determining the composition of the soil microbe community. Moreover, N addition affected both bacterial diversity and community composition more than those of fungi in subtropical forests.

Keywords: Available N; DOC; Nitrogen addition; soil microbial community

Introduction

Industrialization and urbanization have caused an increase in the quantity of nitrogen and phosphorus entering terrestrial systems [1], especially in China’s warm and humid climatic zone [2]. Annually, atmospheric N deposition in China increased from 7.6 to 20 Tg in 1978−2010 [3], which focused the attention of researchers in the effects of N deposition on terrestrial ecosystem. Excessive N input causes various changes. In particular, it has several negative effects on the structure and functioning of terrestrial ecosystems, such as soil acidification [4, 5], a decline in tree productivity [6, 7, 8], a reduction in plant diversity [9, 10], and changes in understory vegetation composition [11, 12]. It also causes alterations in ectomycorrhizal fungal communities [13] and soil bacterial communities [14, 15].

Soil bacteria and fungi play pivotal roles in terrestrial ecosystems, including mediation of biogeochemical processes (e.g., carbon and nitrogen cycling) [14, 16] and promotion of above-ground plant health and productivity [2, 17, 18]. Until now, there have been 10 meta-analyses studying the effects of N addition on soil microbial biomass and community composition [19-28]. In summary, simulated N deposition generally suppressed microbial biomass [2], but the effects may vary among biome types. Nitrogen addition reduced soil microbial biomass carbon (MBC) in temperate forests and grasslands, but significantly increased MBC in tropical/subtropical forests [28]. Additionally, N addition is supposed to increase microbial biomass via increasing the C or/and N resource availability [27].

In previous meta-analysis, N addition increased the Shannon indices, reduced the

bacterial Chao1 indices, and the effect on soil bacterial richness was larger than that on fungal richness [28]. Simulated N addition in a subtropical forest significantly decreased microbial diversity, with variations in the predominant microbial life-history, and enriched the copiotrophic microbial community [29]. Liu et al. [30] showed that N addition significantly increased the fungi/bacteria ratio (F/B) in the tropical forest, whereas Li et al. [31] found that short-term N addition did not alter F/B in the soil. In contrast, Tian et al. [32] reported that N enrichment decreased F/B in the subtropical forest. Within a soil bacterial or fungal community, different taxonomic or functional bacterial or fungal groups may also exhibit diverse responses. It has been hypothesized that elevated N enrichment should decrease the abundance of oligotrophic taxa but increase the abundance of copiotrophic taxa, i.e., copiotrophic hypothesis [14, 15, 29, 33-35].

Meanwhile, several potential driving factors were proposed to account for shifts in sensitive microbial communities in some ecosystems [5]. Among them, soil pH is considered to be a powerful predictor of soil bacterial community composition at the continental scale [35, 36]. However, fungal community composition is less influenced by soil pH because fungi generally exhibit larger pH ranges for optimum growth than those of bacteria [36]. Furthermore, soil microbial communities were positively correlated with soil carbon and nitrogen concentrations [38]. Nitrogen addition could directly influence soil bacterial community composition by changing the nitrogen availability, e.g., modified ammonium N (NH_4^+-N) concentration [5, 27, 39]. Soil physicochemical characteristics govern the diversity and community structure of microorganisms [40]. Soil bacterial

community composition is closely related to soil NH_4^+-N content in tropical forest soil under N enrichment owing a narrow pH decrease in severely acidic soil (pH < 4.5) [5]. Although these mechanisms have been researched before, comprehensive research about the different regulatory pathways of N addition on the bacterial and fungal community structure in the forest ecosystem is still limited.

Different measures have been taken to improve the soil quality and protect and improve forest productivity, with fertilization being the most effective and feasible method [41]. Several field studies focused on the effects of nutrients on C and N retention in forest soils [42, 43]， but few studies have investigated the microbial characteristics of underground soil. Subtropical forests in southern China experience extensive N deposition, accompanied by signs of N saturation, such as soil acidification [44]. Therefore, to better understand the responses of bacterial and fungal communities to N addition and their potential mechanisms, we conducted a N addition experiment in this area, specifically raising the following scientific questions: (1) How do bacterial and fungal groups and community structures respond to N addition? (2) How do soil biotic and abiotic factors regulate the responses of bacterial and fungal taxa and community structures to N additions? (3) What are the mechanisms behind potential shifts in bacterial and fungal taxa and community structures in response to N addition?

Materials and Methods

Site description and experimental design

This study was conducted at the Daiyun Mountain Nature Reserve located in Fujian Province, southeast of China (118° 05´ E, 25° 40´ N). The reserve is located in the transition zone from central subtropical to southern subtropical, it is the nature reserve with the highest degree of biodiversity per unit area in China, with the southernmost distribution, and concentrates the largest area of best preserved natural Pinus taiwanensis community (Fig. 1). Pinus taiwanensis is a unique alpine tree species in China that plays a valuable role in ecological restoration. This area is also the largest germ plasm gene pool of Pinus taiwanensis in China, mainly distributed at an altitude of 1000−1800 m. The climate type is subtropical maritime monsoon climate, with cold winter and hot summer, simultaneous rain and heat, and it is humid and rainy, with four distinct seasons, mean annual temperature of 20 °C, mean annual precipitation of 1800 mm, and 80% relative humidity. The soil is an Ultisol formed from sandstone and classified as red soil by the Chinese soil classification. Total N deposition in this region is about 38 kg N ha^{-1} yr^{-1} [44].

In the Pinus taiwanensis forest, we randomly chose experimental plots to perform the simulated N addition treatment in May 2018. In each experimental plot , 12 subplots (10 × 10 m) were randomly placed for three levels of N addition, including control (CT), low nitrogen (LN), and high nitrogen addition (HN) (0, 40, and 80 kg N ha^{-1} yr^{-1}, respectively), with four replicates for each application level. Each plot was surrounded by a 5-m wide buffer zone. We used urea [$CO(NH_2)_2$] as nitrogen source added into the experimental plots from March to September every year. The required amount of urea [$CO(NH_2)_2$] was dissolved in 8 L purified water, and CT plots received an equivalent volume of water

without urea.

Soil sampling and analysis

In May 2019, soil samples were randomly collected from 5−8 cores at depths of 0−10 (topsoil; horizon A) and 10−20 cm (subsoil; horizon B) in each plot after removing the surface litterfall. Soil samples were passed through a 2-mm sieve after removing the litter, roots, and stones, and divided in three parts. One portion of each sample was immediately processed to measure soil moisture content (SMC), mineral N, dissolved organic C (DOC), and stored at -80 °C for soil molecular biological measurement. Another portion was air-dried for soil physicochemical parameters analysis. The last portion was passed through a 0.149-mm sieve to measure total soil C and N.

Soil pH was determined using a glass electrode (STARTER 300; OHAUS, USA). Samples were shaken for 0.5 h in a 1:2.5 soil:water solution (w/v). Soil moisture content was assessed by oven drying for 48 h at 105 °C to constant mass. The soil total N to organic C (SOC) ratio (C:N) was measured using an elemental analyzer (Elementar Vario EL III; Elementar, Langenselbod, Germany). After 2 M KCl-extracts of fresh soil sample, the soil suspensions were centrifuged at 4000 rpm for 10 min, filtered through Whatman 42 filter papers, and analyzed for exchangeable NH_4^+-N and NO_3^--N with a Continuous Flow Analytic System (Skalar san++; Skalar, Breda, the Netherlands). DOC was extracted with deionized water in a 1:4 soil:water solution (w/v) by shaking for 0.5 h, centrifuging at 4000 rpm for 0.5 h, and then filtering through 0.45-μm Millipore filters. The extracts were

analyzed on a TOC-VCPH/CPN analyzer (Elementar Analysensysteme GmbH, Germany) to determine the DOC concentrations.

Soil microbial biomass carbon (MBC) and nitrogen (MBN) were measured using the chloroform-fumigation extraction method [45]. MBC correction factor was 0.38 [46], while that of MBN was 0.45 [47].

Quantitative PCR (qPCR) analysis of soil microbial abundance

Soil DNA was extracted from 0.25 g soil samples using PowerSoil DNA Isolation Kit (MoBio Laboratories, Carlsbad, CA) following the manual. Purity and quality of genomic DNA were checked on 0.8% agarose gels. The quantitative PCR (qPCR) method measured the abundance of the soil bacteria 16S rDNA gene and fungi ITS which were amplified with the primers detailed in Table S1 [48].

For each soil sample, a 10-digit barcode sequence was added to the 5´ end of the forward and reverse primers (provided by Allwegene Company, Beijing). PCR was carried out on a Mastercycler Gradient (Eppendorf, Germany) using 25 μl reaction volumes, containing 12.5 μl 2× Taq PCR MasterMix, 3 μl BSA (2 ng μl^{-1}), 2 Primer (5 μM), 2 μl template DNA, and 5.5 μl ddH_2O. Cycling parameters were 94 °C for 5 min, followed by 30 cycles of 94 °C for 30 s, 55 °C for 30 s, and 72 °C for 30 s, with a final extension at 72 °C for 10 min. Three PCR products per sample were pooled to mitigate reaction-level PCR biases. PCR products were purified using a QIAquick Gel Extraction Kit (QIAGEN, Germany) and quantified using real time PCR. The fungal-to-bacterial (F/B) ratio was

calculated using the 16S rDNA and ITS gene copy numbers.

Illumina Miseq sequencing and bioinformatics analysis

Deep sequencing was performed on Miseq platform at Allwegene Company (Beijing). After the run, image analysis, base calling, and error estimation were performed using Illumina Analysis Pipeline Version 2.6.

Raw data were first screened, and sequences were removed from consideration if they were shorter than 200 bp, had a low quality score (≤ 20), contained ambiguous bases, or did not exactly match to primer sequences and barcode tags. Qualified reads were separated using the sample-specific barcode sequences and trimmed with Illumina Analysis Pipeline Version 2.6. Then, the dataset was analyzed using QIIME (v1.8.0). Sequences were clustered into operational taxonomic units (OTUs) at a similarity level of 97% [49] to generate rarefaction curves and to calculate the richness and diversity indices. The ribosomal database project classifier tool was used to classify all sequences into different taxonomic groups [50].

Statistical analysis

All data were presented as means in the tables and figures. Statistical analyses were performed using SPSS 20.0 (SPSS, Inc., Chicago, IL, USA), and data were tested for normality and homoscedasticity before statistical analyses. One-way analysis of variance (ANOVA) was used to determine the differences in soil pH, soil microbial biomass, and

multiple comparisons were conducted using a least significant difference (LSD) test at a significance level of $p < 0.05$. Moreover, correlation analysis was performed with the Pearson test (two-tailed) at two significance levels, i.e., $p < 0.05$ and $p < 0.01$.

To examine the similarity between different samples, we used clustering and PCA based on the OTU information from each sample R [51]. The evolutionary distances between microbial communities from each sample were calculated using the tayc coefficient and represented as an Unweighted Pair Group Method with Arithmetic Mean (UPGMA) clustering tree describing the dissimilarity (1 - similarity) between multiple samples [52]. Multivariate statistical analysis, known as LDA (linear discriminant analysis) Effect Size, was performed to find the species with significant differences in abundance in the soil samples.

Differences in soil properties and microbial parameters were assessed by one-way ANOVA, followed by LSD's post hoc tests. For the soil microbial α-diversity levels, the relative abundances of the bacterial or fungal communities and the F/B ratio were analyzed using one-way ANOVA. Two-way ANOVA was used to compare the soil fractions (topsoil and subsoil) and fertilizer treatments. Pearson's correlation analyses were performed to assess the relationships between soil properties and microbial α-diversity indices. The Chao1, Simpson, and Shannon α-diversity levels; phylogenetic diversity whole tree (PD whole tree); and observed species indices were calculated using QIIME software (version 1.8.0). Partial Least Squares Discrimination Analysis (PLS-DA), which is a multivariate statistical analysis method for discriminant analysis, showed the β-diversity in soil bacterial

and fungal community with N additions. Pearson correlation analysis was used to address the relationships between soil properties, microbial abundance, and microbial biomass. In all tests, p-value < 0.05 was considered statistically significant. Redundancy analysis (RDA) was used to identify the relationship between changes in the soil microbial community composition and soil environmental factors and was performed using CANOCO 5.0 (Ithaca, NY, USA).

Results

Response of soil properties and microbial biomass to N additions

Among the studied soil chemical properties, soil H_2O-extractable pH, SMC, CEC, SOC, total N (TN), total P (TP), and C:N were not affected by N addition (Table 1). Soil KCl-extractable pH increased with N addition to the subsoil, whereas the pH of the topsoil was not affected. Contrastingly, soil NO_3^--N concentration significantly increased with N addition in topsoil, and soil NH_4^+-N concentration significantly decreased with N addition in both topsoil and subsoil. Moreover, N addition reduced soil available N and DOC concentration in both topsoil and subsoil. In addition, MBN differences with N addition were only observed in topsoil, and N addition did not affect MBC in both topsoil and subsoil.

Effects of N addition on bacterial and fungal gene copy numbers

Bacterial and fungal abundances were determined by qPCR. The peak number of 16S

rDNA gene copies appeared at LN plots in both topsoil and subsoil, and the minimum appeared at HN plots in both topsoil and subsoil (Table S2). In subsoil, bacterial abundance in LN and HN plots was slightly different. In contrast, N addition did not have a significant effect on bacterial abundance in the topsoil ($p = 0.76$). Additionally, a significantly positive correlation was observed between bacterial abundance and KCl-extractable pH ($R^2 = 0.78$, $p < 0.01$) (Table S3). The peak number of ITS gene copies occurred at LN plots in topsoil and CT plots in subsoil, and the minimum occurred at HN plots in both topsoil and subsoil (Table S2). Unlike bacterial abundance, N additions had no significant effect on fungal abundance.

F/B values were greater in 0−10 cm soil samples than in 10−20 cm soil samples (Fig. S2). Additionally, they were positively correlated with soil MBN ($R^2 = 0.58$, $p < 0.01$) and NO_3^--N ($R^2 = 0.51$, $p < 0.05$) levels and negatively correlated with soil pH (H_2O) values ($R^2 = 0.61$, $p < 0.01$) (Table S3).

Effects of N addition on bacterial and fungal diversity

Overall, 3760 OTUs were obtained from 1185330 high quality and chimera-free clean tags by Hiseq sequencing of 16S rDNA gene amplicons, with an average of 19719−89416 clean tags per sample, and 4584 OTUs were obtained from a total of 1171127 high quality and chimera-free clean tags by Hiseq ITS sequencing, with an average of 23183−86895 clean tags per sample. The rarefaction curves showed these as reasonable amounts of sequencing data (Figs. S1A & B) with Good's coverage of 97.13%−98.75%, and indicated

sufficient sequence reads to capture the bacterial and fungal α-diversity (Table S4).

Soil bacterial and fungal α-diversity were affected differently by N addition in topsoil and subsoil (Table 2; Table S5). Nitrogen addition decreased bacterial α-diversity (Chao1, Observed species, PD whole tree, Shannon indices) in subsoil but did not affect it significantly in topsoil (Table S5). However, topsoil bacterial richness indices in LN, including Chao1, Observed species, and PD whole tree, were all slightly higher than those in other treatments, although the difference was not significant ($p > 0.05$) (Table S5). The fungal α-evenness (Shannon, Simpson) and richness indices (Chao1 and PD whole tree), which were significantly higher in the topsoil than in the subsoil, decreased and increased with N addition in topsoil and subsoil, respectively (Table S5).

Pearson's correlation coefficients revealed a significant correlation between soil microbial α-diversity and soil properties (Figs. 2A & B). Changes in soil bacterial α-diversity (Shannon and Simpson index) induced by N addition were most closely related to soil pH and NO_3^--N, especially NO_3^--N with positive correlation. Changes in soil bacterial α-richness (Chao1 index) were most closely related to soil pH and SOC with negative and positive correlation, respectively. However, it was weakly correlated with C:N, CEC, TP, and available N.

Moreover, regarding the distance among multiple samples, i.e., β-diversity, PLS-DA showed that soil bacterial and fungal communities differed significantly among N addition treatments (Figs. S2A, B, C & D).

Relative abundance of dominant microbial taxa and species variation

The relative abundance of dominant phyla (bacterial and fungal) in topsoil and subsoil were shown in Fig. 3, and bacterial and fungal species variation with different treatments analyzed by cladogram of Lefse analysis were shown in Fig. S3.

With LN, the bacterial phyla of Proteobacteria, Acidobacteria, and Actinobacteria occupied 80.25% and 74.22% of the total sequences in the topsoil and subsoil, respectively, followed by Firmicutes (3.47% and 2.2%, respectively), Planctomycetes (4.3% and 6.62%, respectively), and Chloroflexi (3.23% and 8.24%, respectively). With HN, the bacterial phyla of Proteobacteria, Acidobacteria, and Actinobacteria occupied 76.95% and 77.25% of the total sequences in the topsoil and subsoil, respectively, followed by Firmicutes (4.89% and 0.74%, respectively), Planctomycetes (4.22% and 5.97%, respectively), and Chloroflexi (2.87% and 9.62%, respectively) (Fig. 3). For fungi, the phyla Ascomycota was the most abundant with LN, occupying 46.9% and 43.29% in topsoil and subsoil, respectively. Basidiomycota (41.8% and 37.5%, respectively) were also present mostly in both soils. With HN, Ascomycota (50.09% and 39.59%, respectively) and Basidiomycota (37.5% and 51.65%, respectively) were most abundant in topsoil and subsoil (Fig. 3).

Regarding the bacteria, the relative abundance of the phyla Firmicutes and Chloroflexi increased with N addition, especially HN. At the class level, there were six species varying with N addition, i.e., Subgroup 2 and Acidobacteria from phylum Acidobacteria; Ktedonobacteria and JG37-AG-4 from phylum Chloroflexi, Clostridia from phylum Firmicutes, and Acidimicrobiia from phylum Actinobacteria (Fig. S3A).

For the fungi, there were two families with different abundance levels after N addition treatment in both soil layers. One was the family Myxotrichaceae from phylum Ascomycota, and the other was unidentified. At the genus level, the relative abundances of Oidiodendron and Cortinarius belonging to the Ascomycota and Basidiomycota phyla, respectively, were significantly decreased with HN in both soil layers (Fig. S3B).

Bacterial and fungal community structure and correlations with environmental parameters

Two-dimensional RDA was carried out at the OTU level (Fig. 4) and indicated that soil DOC was the most important factor influencing soil bacterial community composition, and their relationship was closely correlated with the first RDA axis (Fig. 4A).

Regarding fungal community structure (Fig. 4B), RDA revealed that these soil variables explained 45.8% of the variation in the fungal community by the first two constrained axes, with the first and second axis explaining 29.17% and 16.63%, respectively. Moreover, RDA clearly showed that the soil-available N and MBN concentrations were the most significant contributors to the variation in fungal communities ($p < 0.01$; Table S6).

Discussion

Effect of nitrogen addition on bacterial and fungal gene abundance and diversity

Previous studies showed that N additions exerted negative [53, 54], neutral [45, 55],

or positive [56, 57] effects on microbial biomass CN (MBC/MBN) in tropical/subtropical forests, and several studies from tropical/subtropical forests observed increases in F/B under short-term N additions [30, 57, 58]. However, recent meta-analysis found that N additions increased MBC and decreased F/B in tropical/subtropical forests [27]. Overall, MBN was significantly increased by N addition (Table 1). As soil microbes are mainly C limited [59], the increase of labile C input is expected to multiply microbial biomass.

Previous studies have demonstrated that N additions decrease microbial abundance and diversity [5, 24, 28, 35, 60]. Contrastingly, bacterial and fungal abundance in our study were increased by LN, although without statistical significance. However, HN decreased the abundance of bacterial 16S rDNA gene and fungal ITS gene, agreeing with recent studies [28, 61, 62]. Particularly, Pearson's correlation analysis in this study showed that the abundance of bacterial 16S rDNA gene as well as F/B were strongly correlated with soil pH, indicating that soil pH was a decisive factor affecting them (Table S3). Furthermore, F/B was correlated with soil properties including NO_3^-, TN, SOC, and microbial biomass. Moreover, bacterial abundance was significantly influenced by soil pH because most bacterial taxa showed relatively narrow growth tolerances, especially within 4−7 pH [28, 37, 63]. Moreover, fungal abundance was correlated to CEC (Table S3).

Soil properties play vital roles in shaping soil bacterial diversity in terrestrial ecosystems, which is drastically affected by C and N supply to the soil [64, 65]. Among the analyzed soil chemical factors, pH was the factor that most strongly influenced soil bacterial diversity (Fig. 2A). It has been noted that soil pH controls microbial diversity in

ecosystems [33, 39, 66, 67]. Furthermore, available N significantly affected soil bacterial diversity. In this study, the abundance of bacterial 16S rDNA gene was closely related to available N, further confirming that N availability was a crucial factor altering bacterial abundance [5, 68, 69].

Influence of N addition on bacterial and fungal community structure

In this study, high-throughput sequencing analysis indicated that Proteobacteria, Acidobacteria, Actinobacteria and Chloroflexi were the predominant bacterial phyla in the acidic forest soil, which was in agreement with previous studies [5, 70]. In topsoil, the relative abundance of Proteobacteria and Actinobacteria decreased with LN and HN, while that of Acidobacteria and Chloroflexi increased. These findings were against the copiotrophic hypothesis proposed by Fierer et al. [69]. However, our findings in subsoil (10−20 cm) supported the copiotrophic hypothesis. Moreover, there were no significant differences in the abundance of Proteobacteria, Acidobacteria, Actinobacteria, Chloroflexi, Planctomycetes, Firmicutes, and Verrucomicrobia between LN and HN treatments. The reason may be that soil pH was 4.22−4.35 in topsoil (Table 1). Among various soil properties influencing soil microbial communities, pH has widely been recognized as a key factor, especially in subtropical regions with acidic soil, because most microbes are inhibited when pH is below 4.5 [71-73].

Proteobacteria and Actinobacteria have been reported to be related to superior carbon availability, and they exhibit relatively rapid growth rates. Meanwhile, Acidobacteria are

oligotrophic bacteria found in nutrient deficient and very acidic environments, and have the ability to degrade complex and recalcitrant carbon compounds [74]. Zhang et al. [64] found relatively large abundance of Acidobacteria under very low SOC (2.66 g kg^{-1}). In addition, Acidobacteria have been declared to be negatively correlated with soil C availability [34]. The class Ktedonobacteria comprises a unique group of prokaryotes known for their actinomycetes-like morphology, which are possible C-cycling-related bacterial taxa. It was first reported by Cavaletti et al. [75], and was placed in the phylum Chloroflexi [76], generally known to be a deep-branching lineage of the prevailing bacteria. The class Acidimicrobiia belongs to the phylum Actinobacteria, which play a prominent role in soil C, N, and P cycling by producing extracellular enzymes and symbiosis with plants [77-79]. This extracellular enzymes break down litter, further altering the soil C fraction [80]. Several bacterial strains from Actinobacteria that are capable of fixing N and removing P have been isolated [80-82]. Several functions relating to carbohydrate, amino acid, xenobiotics, and energy metabolism declined in samples receiving N addition [69].

Nitrogen addition may affect bacteria more than it affects fungi, maybe owing to bacteria having more copiotrophic characteristics [15]. Soil fungal community is a functionally diverse group [83] that mediates many ecological processes and affects plant growth and soil health [84]. ITS sequencing analysis indicated that Ascomycota and Basidiomycota were the dominant fungal phyla in the acidic forest soil, which supported the copiotrophic hypothesis. Because soil nutrients decrease with soil depth, the relative abundance in topsoil of Basidiomycota, which is the representative phylum of oligotrophic

taxa, was lower than that of Ascomycota (Fig. 3). Previous studies have shown that N addition increases the relative abundance of copiotrophic phylum Ascomycota and decreased that of oligotrophic phylum Basidiomycota according to the copiotroph-oligotroph concept [34, 86-88]. The family Myxotrichaceae and genus Oidiodendron are related to the decomposition of cellulose. Although no significant effect of N addition on the fungal community composition was observed (Fig. 3), RDA analysis indicated that fungal community structure was correlated with soil N availability (NH_4^+ and NO_3^-, Fig. 4; Table S6), suggesting that N addition was likely to affect species variation directly via increasing N availability. Overall, the limited responses of fungal diversity, richness, and community composition to N addition in our study may be caused by increased N loss from soil through denitrification and leaching [88].

RDA analysis suggested a significant relationship between soil bacterial community composition and DOC concentration rather than soil pH in the subtropical acidic forest soil (Fig. 4A, Table S6). As far as we know, DOC is the preferred nutrient element for most bacteria. Our results showed that soil MBN, along with soil NO_3^- and available N, are key factors for forecasting dominant bacterial community composition using RDA. Urea is the principal mode of nitrogen application, and its hydrolysis can consume H^+ and rise pH. Mostly, NH_4^+ is formed after urea is applied to the soil, which counteracts the H^+ enrichment caused by NO_3^- leaching, and may be the reason why soil pH has not changed after short-term nitrogen application in our study. Nitrogen addition could influence soil microbes directly, by changing soil available N content, or indirectly, by changing soil C

availability, C:N, and soil pH [19, 89]. As N addition promotes the consumption of C, C supply becomes unstable and lignin decomposition is reduced [90], which leads to a decline in C reserves for other heterotrophic microbial metabolic activities [91]. According to the resource allocation theory, sufficient N supply can stimulate C-related microbial growth, leading to faster decomposition of cellulose-rich litter [92, 93]. Given the N saturation hypothesis, N addition may reduce the microbial requirements for additional N in all ecosystems, and C or P limitation of soil microbes are expected to be motivated or aggravated [94-96]. RDA analysis showed that fungal community composition was correlated with soil available N ($p < 0.01$) (Fig. 4B, Table S6), which suggests that fungi-dominated niches led to faster mineralization of N as well as a slightly lower microbial retention of available N (NH_4^+) [97, 98].

In this study, PLS-DA demonstrated that bacteria and fungi occupied different ecological niches and developed independently (Fig. S2). We believe that an increase in available C and N, instead of soil pH alterations, caused the change in microbial diversity under N addition. This finding is consistent with the latest studies reporting that nutrient availability controlled the responses of the soil-microbe system to N addition [24, 25, 99].

Conclusion

This study in conjunction with other studies showed that the soil bacterial and fungal diversity and community structure were weakly influenced by short-term N addition. Nitrogen addition reduced soil bacterial community diversity in subsoil and resulted in an increased abundance of oligotrophic bacterial communities in severely acidic tropical

forest soil. Interestingly, N addition had positive effects on some C-cycling-related bacterial taxa (e.g., Ktedonobacteria and Acidobacteria). Microbial community composition correlated with soil characteristics, especially organic carbon, available nitrogen, NH_4^+, and NO_3^-. Soil microbes remain critical regulators in biogeochemical cycling and can transform soil nutrient elements (non-available nitrogen and carbon) into usable forms (available nitrogen and carbon. Nitrogen addition affected microbial communities mainly through increasing nutrient availability rather than through soil acidification. Change in N addition is a long and gradual process, and its influence on the soil-microbe system may shift over time. Hence, the interactive effect of long-term N addition on the biogeochemical cycling functions of these subtropical forest ecosystems should be the subject of future studies.

Author Contributions: Conceived and designed the experiments: JYC, YMC, JGX, KML. Performed the experiments: JYC, XCY, YW, LC, QXZ, KCM. Analyzed the data: JYC, XCY, YZZ, JCZ. Wrote the paper: JYC. Reviewed the manuscript: QFZ, YMC.

Funding: The research was supported by the National Natural Science Foundation of China (No. 31670620).

Acknowledgments: We thank Prof. Zhigao Sun and Prof. Quanlin Zhong for encouraging the development of this study and to Yating Chen and Liangtai Zhou for their help in field

work.

Conflicts of Interests: The authors declare no conflict of interest.

Reference:

1. Shukla, P.R.; Skea, J.; Calvo Buendia, E.; Masson-Delmotte, V.; Pörtner, H.O.; Roberts, D.C.; Zhai, P.; Slade, R.; Connors, S.; van Diemen, R.; Ferrat, M.; Haughey, E.; Luz, S.; Neogi, S.; Pathak, M.; Petzold, J.; Portugal Pereira, J.; Vyas, P.; Huntley, E.; Kissick, K.; Belkacemi, M.; Malley, J. Land–climate interactions. In: Climate Change and Land: an IPCC special report on climate change, desertifcation, land degradation, sustainable land management, food security, and greenhouse gas fluxes in terrestrial ecosystems. 2019.
2. Xie, L.; Zhang, Q.; Cao, J.; Liu, X.; Xiong, D.; Kong, Q.; Yang, Y. Effects of warming and nitrogen addition on the soil bacterial community in a subtropical Chinese Fir plantation. Forests 2019; 10: 861.
3. Cui, S.; Shi, Y.; Groffman, P.M.; Schlesinger, W.H.; Zhu, Y. Centennial-scale analysis of the creation and fate of reactive nitrogen in China (1910–2010). Proc. Natl. Acad. Sci. USA. 2013; 110: 2052–2057.
4. Lu, X.; Mao, Q.; Gilliam, F.S.; Luo, Y.; Mo, J. Nitrogen deposition contributes to soil acidification in tropical ecosystems. Glob. Chang. Biol. 2014; 20: 3790–3801.
5. Nie, Y.; Wang, M.; Zhang, W.; Ni, Z.; Hashidoko, Y.; Shen, W. Ammonium nitrogen

content is a dominant predictor of bacterial community composition in an acidic forest soil with exogenous nitrogen enrichment. Sci. Total Environ. 2018; 624: 407–415.

6. De Vries, W.; Posch, M.; Simpson, D.; Reinds, G.J. Modelling long-term impacts of changes in climate, nitrogen deposition and ozone exposure on carbon sequestration of European forest ecosystems. Sci. Total Environ. 2017; 1097–1116, 605–606.

7. Schmitz, A.; Tanja, G.M.; Sanders; Bolte A.; Bussotti F.; Dirnböck T.; Johnson J.; Peñuelas J.; Pollastrini M.; Prescher A.; Sardans J. et al. Responses of forest ecosystems in Europe to decreasing nitrogen deposition. Environ. Pollut. 2019; 244: 980–994.

8. Giammarchi, F. P., Panzacchi, M.; Tonon, V.G. Tree growth and water-use efficiency do not react in the short term to artificially increased nitrogen deposition. Forests 2020; 11, 47.

9. Stevens, C.J.; Dise, N.B.; Mountford, J.O.; Gowing, D.J. Impact of nitrogen deposition on the species richness of grasslands. Science 2004; 303:1876–1879.

10. Lu, X.; Mo, J.; Gilliam, F.S.; Zhou, G.; Fang, Y. Effects of experimental nitrogen additions on plant diversity in an old-growth tropical forest. Glob. Chang. Biol. 2010; 16: 2688–2700.

11. Dirnböck, T.; Grandin, U.; Bernhardt-Romermann, M.; Beudert, B.; Canullo, R.; Forsius, M.; Grabner, M.T.; Holmberg, M.; Kleemola, S.; Lundin, L.; Mirtl, M.; Neumann, M.; Pompei, E.; Salemaa, M.; Starlinger, F.; Staszewski, T.; Uzieblo, A.K. Forest floor vegetation response to nitrogen deposition in Europe. Glob. Chang. Biol. 2014; 20: 429–440.

12. van Dobben, H.F.; De Vries, W. The contribution of nitrogen deposition to the eutrophication signal in understorey plant communities of European forests. Ecol. Evol. 2017; 7: 214–227.
13. van der Linde, S.; Suz, L.M.; Orme, C.D.L.; Cox, F.; Andreae, H.; Asi, E.; Atkinson, B.; Benham, S.; Carroll, C.; Cools, N.; et al. Environment and host as large-scale controls of ectomycorrhizal fungi. Nature 2018; 558: 243–248.
14. Fierer, N.; Lauber, C.L.; Ramirez, K.S.; Zaneveld, J.; Bradford, M.A.; Knight, R. Comparative metagenomic, phylogenetic and physiological analyses of soil microbial communities across nitrogen gradients. ISME J. 2012; 6: 1007–1017.
15. Ramirez, K.S.; Craine, J.M.; Fierer, N. Consistent effects of nitrogen amendments on soil microbial communities and processes across biomes. Glob. Chang. Biol. 2012; 18: 1918–1927.
16. López-Lozano, N.E.; Heidelberg, K.B.; Nelson, W.; Garcia-Oliva, F.; Eguiarte L.E. Microbial secondary succession in soil microcosms of a desert oasis in the Cuatro Cienegas Basin, Mexico. Peer J. 2013; 1–47.
17. Van Der Heijden, M.G.; Bardgett, R.D.; Van Straalen, N.M. The unseen majority: soil microbes as drivers of plant diversity and productivity in terrestrial ecosystems. Ecol. Lett. 2008; 11: 296–310.
18. Zhou, J.; Liu, X.; Xie, J.; Lyu, M.; Zheng, Y.; You, Z.; Fan, Y.; Lin, C.; Chen, G.; Chen, Y.; Yang, Y. Nitrogen addition affects soil respiration primarily through changes in microbial community structure and biomass in a subtropical natural forest. Forests

2019; 10, 435.

19. Treseder, K.K. Nitrogen additions and microbial biomass: a meta-analysis of ecosystem studies. Ecol. Lett. 2008; 11: 1111–1120.

20. Janssens, I.A.; Dieleman, W.; Luyssaert, S.; Subke, J.A.; Reichstein, M.; Ceulemans, R.; Ciais, P.; Dolman, A.J.; Grace, J.; Matteucci, G.; et al. Reduction of forest soil respiration in response to nitrogen deposition. Na. Geo. 2010; 3: 315–322.

21. Liu, L.; Greaver, T.L. A global perspective on belowground carbon dynamics under nitrogen enrichment. Ecol. Lett. 2010; 13: 819–828.

22. Lu, M.; Zhou, X.H.; Luo, Y.Q.; Yang, Y.H.; Fang, C.M.; Chen, J.K.; Li, B. Minor stimulation of soil carbon storage by nitrogen addition: a meta-analysis. Agr. Ecosyst. Environ. 2011; 140: 234–244.

23. Lu, M.; Yang, Y.H.; Luo, Y.Q.; Fang, C.M.; Zhou, X.H.; Chen, J.K.; Li, B. Responses of ecosystem nitrogen cycle to nitrogen addition: a meta-analysis. New Phytol. 2011; 189: 1040–1050.

24. Geisseler, D., Scow, K.M. Long-term effects of mineral fertilizers on soil microorganismse: a review. Soil Biol. Biochem. 2014; 75: 54–63.

25. Geisseler, D.; Lazicki, P.A.; Scow, K.M. Mineral nitrogen input decreases microbial biomass in soils under grasslands but not annual crops. Appl. Soil Ecol. 2016; 106: 1–10.

26. Yue, K.; Peng, Y.; Peng, C.H.; Yang, W.Q.; Peng, X.; Wu, F.Z. Stimulation of terrestrial ecosystem carbon storage by nitrogen addition: a meta-analysis. Sci. Rep. 2016; 6,

19895.

27. Zhou, Z.; Wang, C.; Zheng, M., Jiang, L.; Luo, Y. Patterns and mechanisms of responses by soil microbial communities to nitrogen addition, Soil Biol. Biochem. 2017; 115: 433–441.

28. Wang, C.; Liu, D.; Edith Bai. Decreasing soil microbial diversity is associated with decreasing microbial biomass under nitrogen addition. Soil Biol. Biochem. 2018; 120: 126–133.

29. Li, H.; Xu, Z.; Yang, S.; Li, X.; Top, E.M.; Wang, R.; Zhang, Y.; Cai, J.; Yao, F.; Han, X.; Jiang, Y. Responses of soil bacterial communities to nitrogen deposition and precipitation increment are closely linked with aboveground community variation. Microb. Ecol. 2016; 71: 974–989.

30. Liu, L.; Zhang, T.; Gilliam, F.S.; Gundersen, P.; Zhang, W.; Chen, H.; Mo, J. Interactive effects of nitrogen and phosphorus on soil microbial communities in a tropical forest. PLoS One 2013; 8, e61188.

31. Li, J.; Li, Z.; Wang, F.; Zou, B.; Chen, Y.; Zhao, J.; Mo, Q.; Li, Y.; Li, X.; Xia, H.; Effects of nitrogen and phosphorus addition on soil microbial community in a secondary tropical forest of China. Biol. Fert. Soils, 2015; 51: 207–215.

32. Tian, D.; Jiang, L.; Ma, S.; Fang, W.; Schmid, B.; Xu, L.; Zhu, J.; Li, P.; Losapio, G.; Jing, X. et al. Effects of nitrogen deposition on soil microbial communities in temperate and subtropical forests in China. Sci. Total Environ. 2017; 607: 1367–1375.

33. Ramirez, K.S.; Lauber, C.L.; Knight, R.; Bradford, M.A.; Fierer, N. Consistent effects

of nitrogen fertilization on soil bacterial communities in contrasting systems. Ecology, 2010; 91: 3463–3470.

34. Fierer, N.; Bradford, M.A.; Jackson, R.B. Toward an ecological classification of soil bacteria. Ecology 2007; 88: 1354–1364.

35. Ling, N.; Chen, D.; Guo, H.; Wei, J.; Bai, Y.; Shen, Q.; Hu, S. Differential responses of soil bacterial communities to long-term N and P inputs in a semi-arid steppe. Geoderma, 2017; 292: 25–33.

36. Lauber, C.L.; Hamady, M.; Knight, R.; Fierer, N. Pyrosequencing-based assessment of soil pH as a predictor of soil bacterial community structure at the continental scale. Appl. Environ. Microb. 2009; 75 (15): 5111–5120.

37. Rousk, J.; Bååth, E.; Brookes, P.C.; Lauber, C.L.; Lozupone, C.; Caporaso, J.G.; Knight, R.; Fierer, N. Soil bacterial and fungal communities across a pH gradient in an arable soil. ISME J. 2010; 4 (10): 1340–1351.

38. Stone, M.M.; Kan, J.; Plante, A.F. Parent material and vegetation influence bacterial community structure and nitrogen functional genes along deep tropical soil profiles at the Luquillo Critical Zone Observatory. Soil Biol. Biochem. 2015; 80: 273–282.

39. Zeng, J.; Liu, X.; Song, L.; Lin, X.; Zhang, H.; Shen, C.; Chu, H. Nitrogen fertilization directly affects soil bacterial diversity and indirectly affects bacterial community composition. Soil Biol. Biochem. 2016; 92: 41–49.

40. Albornoz, F. E.; Teste, F. P.; Lambers, H.; Bunce, M.; Murray, D. C.; White, N. E.; & Laliberte, E. Changes in ectomycorrhizal fungal community composition and

declining diversity along a 2 - million - year soil chronosequence. Mol.Ecol. 2016; 25: 4919–4929.

41. Zhang, X. Q.; Kirschbaum, M. U.; Hou, Z.; and Guo, Z. Carbon stock changes in successive rotations of Chinese fir (Cunninghamia lanceolata (lamb) hook) plantations. For. Ecol. Manag. 2004; 202: 131–147.

42. Wei, X.; Blanco, J. A.; Jiang, H.; and Kimmins, J. H. Effects of nitrogen deposition on carbon sequestration in Chinese fir forest ecosystems. Sci. Total Environ. 2012; 416: 351–361.

43. Fan, H. B.; Wu, J. P.; Liu, W. F.; Yuan, Y. H.; Huang, R. Z.; Liao, Y. C., Li, Y. Y. Nitrogen deposition promotes ecosystem carbon accumulation by reducing soil carbon emission in a subtropical forest. Plant Soil. 2014; 379: 361–371.

44. Zhu, J.; He, N.; Wang, Q.; Yuan, G.; Wen, D.; Yu, G.; Jia, Y. The composition, spatial patterns, and influencing factors of atmospheric wet nitrogen deposition in Chinese terrestrial ecosystems. Sci. Total Environ. 2015; 511: 777–785.

45. Turner, BL.; Wright, JS. The response of microbial biomass and hydrolytic enzymes to a decade of nitrogen, phosphorus, and potassium addition in a lowland tropical rain forest. Biogeochemistry, 2014; 117: 115–130.

46. Vance, E.D.; Brookes, P.C.; Jenkinson, D.S. An extraction method for measuring soil microbial biomass C. Soil Biol. Biochem. 1987; 19: 703–707.

47. Brookes, P.C.; Landman, A.; Pruden, G.; Jenkinson, D.S. Chloroform fumigation and the release of soil-nitrogen: a rapid direct extraction method to measure microbial

biomass nitrogen in soil. Soil Biol. Biochem. 1985; 17: 837–842.

48. Munyaka, P. M.; N. Eissa; Bernstein, C. N. Antepartum Antibiotic Treatment Increases offspring susceptibility to experimental colitis: A role of the gut microbiota. PLoS One. 2015; 10 (11): e0142536.

49. Edgar, R.C. UPARSE: Highly accurate OTU sequences from microbial amplicon reads, Nature Methods. 2013.

50. Cole, J. R.; Wang, Q.; Cardenas, E.; Fish, J.; Chai, B.; Farris, R. J.; Kulam-Syed-Mohideen, A. S.; McGarrell, D. M.; Marsh, T.; Garrity, G. M.and Tiedje, J. M. The Ribosomal Database Project: improved alignments and new tools for rRNA analysis. Nucleic Acids Res. 2009; 37: 141–145.

51. Wang, Y.; Sheng, H.; He, Y.; Wu, J.; Jiang, Y.; Tam, N. F.; Zhou, H. Comparison of the levels of bacterial diversity in freshwater, intertidal wetland, and marine sediments by using millions of illumina tags. Appl. Environ. Microbiol. 2012; 78 (23): 8264.

52. Jiang X.T., Peng X., Deng, G.; Sheng, H.; Wang, Y.; Zhou, H.; Tam, N. F. Illumina sequencing of 16S rRNA tag revealed spatial variations of bacterial communities in a mangrove wetland. Microb. Ecol. 2013; 66: 96–104.

53. Peng, Y.; Chen, G.; Chen, G.; Li, S.; Peng, T.; Qiu, X.; Luo, J.; Yang, S.; Hu, T.; Hu, H.; Xu,Z.; Liu, L.; Tang, Y.; Tu, L. Soil biochemical responses to nitrogen addition in a secondary evergreen broad-leaved forest ecosystem. Scientific Reports 2017; 7, 2783

54. Li, Y.; Sun, J.; Tian, D.; Wang, J.; Ha, D.; Qu, Y.; Jing, G.; Niu, S. Soil acid cations induced reduction in soil respiration under nitrogen enrichment and soil acidification.

Sci. Total Environ. 2018; 615: 1535–1546.

55. Fanin, N.; Hättenschwiler, S.; Schimann, H.; Fromin, N.; Bailey, J.K. Interactive effects of C, N and P fertilization on soil microbial community structure and function in an Amazonian rain forest. Funct. Ecol. 2015; 29: 140–150.

56. Li, Y.Q.; Xu, M.; Zou, X.M. Effects of nutrient additions on ecosystem carbon cycle in a Puerto Rican tropical wet forest. Glob. Chang. Biol. 2006; 12: 284–293.

57. Cusack, D.F.; Silver, W.L.; Torn, M.S.; Burton, S.D.; Firestone, M.K. Changes in microbial community characteristics and soil organic matter with nitrogen additions in two tropical forests. Ecology 2011; 92: 621–632.

58. Li, J.; Li, Z.; Wang, F.; Zou, B.; Chen, Y.; Zhao, J.; Mo, Q.; Li, Y.; Li, X.; Xia, H. Effects of nitrogen and phosphorus addition on soil microbial community in a secondary tropical forest of China. Biol. Fertil. Soils 2015; 51: 207–215.

59. Fierer, N.; Strickland, M.S.; Liptzin, D.; Bradford, M.A.; Cleveland, C.C. Global patterns in belowground communities. Ecol. Letters 2009; 12: 1238–1249.

60. Zhou, Z.H.; Wang, C.K. Reviews and syntheses: soil resources and climate jointly drive variations in microbial biomass carbon and nitrogen in China's forest ecosystems. Biogeosciences, 2015; 12: 6751-6760.

61. Wang, Q.; Wang, C.; Yu, W.W.; Turak, A.; Chen, D.; Huang, Y.; Ao, J.; Jiang, Y. and Huang, Z. Effects of Nitrogen and Phosphorus Inputs on Soil Bacterial Abundance, Diversity, and Community Composition in Chinese Fir Plantations, Front. Microbiol. 2018; 9: 1543.

62. Huang, Y.; Kang, R.; Mulder, J.; Zhang, T.; Duan, L. Nitrogen saturation, soil acidification, and ecological effects in a subtropical pine forest on acid soil in southwest China. J. Geophys. Res-Biogeo. 2015; 120: 2457-2472

63. Dai, Z.; Su, W.; Chen, H.; Barberán, A.; Zhao, H.; Yu, M.; Yu, L.; Brookes, PC.; Schadt, CW.; Chang, S. X.; Xu, J. Long-term nitrogen fertilization decreases bacterial diversity and favors the growth of Actinobacteria and Proteobacteria in agro-ecosystems across the globe. Glob. Chang. Biol. 2018; 24 (8): 3452–3461.

64. Zechmeister-Boltenstern, S.; Keiblinger, K.M.; Mooshammer, M.; PENuelas, J.; Richter, A.; Sardans, J.; Wanek, W. The application of ecological stoichiometry to plant–-microbial–soil organic matter transformations. Ecol. Monogr. 2015; 85: 133–155.

65. Zhang, C.; Liu, G.B.; Xue, S.; Wang, G.L. Soil bacterial community dynamics reflect changes in plant community and soil properties during the secondary succession of abandoned farmland in the Loess Plateau. Soil Biol. Biochem. 2016; 97: 40–49.

66. Zhang, X.; Liu, W.; Zhang, G.; Jiang, L.; Han, X. Mechanisms of soil acidification reducing bacterial diversity. Soil Biol. Biochem. 2015; 81: 275–281.

67. Zhang H.; Wang L.; Liu, H.; Zhao, J.; Li, G.; Wang, H.; Lai, X.; Li, J.; Xiu, W.; Yang, D. Nitrogen deposition combined with elevated precipitation is conducive to maintaining the stability of the soil fungal diversity on the Stipa baicalensis steppe. Soil Biol. Biochem. 2018; 117: 135–138.

68. Zhou, J.; Guan, D. W.; Zhou, B. K.; Zhao, B. S.; Ma, M. C.; Qin, J.; Jiang, X.; Chen,

S. F.; Cao F. M.; Shen, D.L.; Li, J. Influence of 34-years of fertilization on bacterial communities in an intensively cultivated black soil in northeast China. Soil Biol. Biochem. 2015; 90: 42–51.

69. Wang, Q. F.; Jiang, X.; Guan, D.; Wei, D.; Zhao, B.; Ma, M.; Chen, S.; Li, L.; Cao, F.; Li, J. Long-term fertilization changes bacterial diversity and bacterial communities in the maize rhizosphere of Chinese Mollisols. Appl. Soil Ecol. 2018; 125: 88–96.

70. Cui, J.; Wang, J.; Xu, J.; Xu, C.; Xu, X. Changes in soil bacterial communities in an evergreen broad-leaved forest in east China following 4 years of nitrogen addition. J. Soils Sediments, 2017; 17: 2156–2164.

71. Högberg, M.N.; Högberg, P.; Myrold, D.D. Is microbial community composition in boreal forest soils determined by pH, C-to-N ratio, the trees, or all three? Oecologia 2007; 150: 590–601.

72. Rousk, J.; Brookes, P.C.; Bååth, E. Fungal and bacterial growth responses to N fertilization and pH in the 150-year 'Park Grass' UK grassland experiment. Fems Microbiol.Ecol. 2011; 76: 89–99.

73. Chen, D. M.; Lan, Z. C.; Bai, X.; Grace, J.B.; Bai, Y. F. Evidence that acidification-induced declines in plant diversity and productivity are mediated by changes in belowground communities and soil properties in a semi-arid steppe. J. Ecol. 2013; 101: 1322–1334.

74. Ai, C.; Liang, G.; Sun, J.; Wang, X.; He, P.; Zhou, W.; He, X. Reduced dependence of rhizosphere microbiome on plant-derived carbon in 32-year long-term inorganic and

organic fertilized soils. Soil Biol. Biochem. 2015; 80: 70–78.

75. Cavaletti, L.; Monciardini, P.; Bamonte, R.; Schumann, P.; Rohde, M.; Sosio, M.; Donadio, S. New lineage of filamentous, spore-forming, gram-positive bacteria from soil. Appl. Environ. Microbiol. 2006; 72: 4360–4369.

76. Yabe, S.; Aiba, Y.; Sakai, Y.; Hazaka, M.; and Yokota, A. Thermosporothrix hazakensis gen. nov., sp. nov., isolated from compost, description of Thermosporotrichaceae fam. nov. within the class Ktedonobacteria Cavaletti et al. 2007 and emended description of the class Ktedonobacteria. Int. J. Syst. Evol. Microbiol. 2010; 60: 1794–1801.

77. Albertsen, M.; Hansen, L.B.S.; Saunders, A.M.; Nielsen, P.H.; Nielsen, K.L. A metagenome of a full-scale microbial community carrying out enhanced biological phosphorus removal. ISME J. 2012; 6: 1094.

78. Sellstedt, A.; Richau, K.H. Aspects of nitrogen-fixing Actinobacteria, in particular free-living and symbiotic Frankia. FEMS Microbiol. Lett. 2013; 342: 179–186.

79. Vetrovsky, T.; Steffen, K.T.; Baldrian, P. Potential of cometabolic transformation of polysaccharides and lignin in lignocellulose by soil Actinobacteria. PLoS One 2014; 9: e0089108

80. Anderson, I.; Abt, B.; Lykidis, A.; Klenk, H.P.; Kyrpides, N.; Ivanova, N. Genomics of aerobic cellulose utilization systems in actinobacteria. PLoS One 2012; 7: e0039331.

81. Pujic, P.; Bolotin, A.; Fournier, P.; Sorokin, A.; Lapidus, A.; Richau, K.H.; Briolay, J.; Mebarki, F.; Normand, P.; Sellstedt, A. Genome sequence of the atypical symbiotic Frankia R43 strain, a nitrogen-fixing and hydrogen-producing actinobacterium.

Genome Announcements 2015; 3: e01387–01315.

82. Nouioui, I.; Ghodhbane-Gtari, F.; del Carmen Monterro-Calasanz, M.; Rohde, M.; Tisa, L.S.; Gtari, M.; Klenk, H.P. Frankia inefficax sp. nov., an actinobacterial endophyte inducing ineffective, non nitrogen-fixing, root nodules on its actinorhizal host plants. Antonie van Leeuwenhoek 2017; 110: 313–320.

83. Paungfoo-Lonhienne, C.; Yeoh, Y.K.; Kasinadhuni, N.R.P.; Lonhienne, T.G.A.; Robinson, N.; Hugenholtz, P.; Ragan, M.A.; Schmidt, S. Nitrogen fertilizer dose alters fungal communities in sugarcane soil and rhizosphere. Scientific Reports 2015; 5: 8678.

84. Klaubauf, S.; Inselsbacher, E.; Zechmeister-Boltenstern, S.; Wanek, W.; Gottsberger, R.; Strauss, J.; Gorfer, M. Molecular diversity of fungal communities in agricultural soils from Lower Austria. Fungal Diversity 2010; 44: 65–75.

85. Chen, W.; Xu, R.; Wu, Y.; Chen, J.; Zhang, Y.; Hu, T.; Yuan, X.; Zhou, L.; Tan, T.; Fan, J. Plant diversity is coupled with beta not alpha diversity of soil fungal communities following N enrichment in a semi-arid grassland. Soil Biol. Biochem. 2018; 116: 388–398.

86. She, W.; Bai, Y.; Zhang, Y.; Qin, S.; Feng, W.; Sun, Y.; Zheng, J.; Wu, B. Resource availability drives responses of soil microbial communities to short-term precipitation and nitrogen addition in a desert shrubland. Front. Microbiol. 2018; 9: 186.

87. Yao, F.; Yang, S.; Wang, Z.; Wang, X.; Ye, J.; Wang, X.; DeBruyn, JM.; Feng, X.; Jiang, Y.; Li, H. Microbial taxa distribution is associated with ecological trophic cascades

along an elevation gradient. Front. Microbiol. 2017; 8: 2071.

88. Niu, S.; Classen, AT.; Dukes, JS.; Kardol, P.; Liu, L.; Luo, Y.; Rustad, L.; Sun, J.; Tang, J.; Templer, PH.; Thomas, RQ.; Tian, D.; Vicca, S.; Wang, Y. P.; Xia, J.; Zaehle, S. Global patterns and substratebased mechanisms of the terrestrial nitrogen cycle. Ecol. Letters 2016; 19(6): 697–709.

89. Serna-Chavez, H.M.; Fierer, N.; van Bodegom, P.M. Global drivers and patterns of microbial abundance in soil. Global Ecol. and Biogeogr. 2013; 22: 1162–1172.

90. Magill, A.H.; Aber, J.D. Long-term effects of experimental nitrogen additions on foliar litter decay and humus formation in forest ecosystems. Plant Soil 1998; 203: 301–311.

91. Zhang, T.; Luo, Y.; Chen, H. Y. H.; Ruan, H. Responses of litter decomposition and nutrient release to N addition: A meta-analysis of terrestrial ecosystems. Appl. Soil Ecol. 2018; 128: 35-42.

92. Allison, SD.; Weintraub, MN.; Gartner, TB.; Waldrop, MP. Evolutionary-economic principles as regulators of soil enzyme production and ecosystem function. In: Shukla G,Varma A (eds) Soil enzymology, 1st edn. Springer, Berlin, 2011; pp 229–243.

93. Carreiro, MM.; Sinsabaugh, RL.; Repert, DA.; Parkhurst, DF. Microbial enzyme shifts explain litter decay responses to simulated nitrogen deposition. Ecology 2000; 81, 2359–2365.

94. Aber, J.D.; Nadelhoffer, K.J.; Steudler, P.; Melillo, J.M. Nitrogen saturation in northern forest ecosystems. Bioscience 1989; 39: 378–386.

95. Gress, S.E.; Nichols, T.D.; Northcraft, C.C.; Peterjohn, W.T. Nutrient limitation in soils

exhibiting differing nitrogen availabilities: what lies beyond nitrogen saturation? Ecology 2007; 88: 119.

96. Kopáček, J.; Cosby, B.; Evans, C.; Hruška, J.; Moldan, F.; Oulehle, F.; Šantrůčková, H.; Tahovská, K.; Wright, R. Nitrogen, organic carbon and sulphur cycling in terrestrial ecosystems: linking nitrogen saturation to carbon limitation of soil microbial processes. Biogeochemistry 2013; 115: 33–51.

97. Rousk, J.; Frey, S.D. Revisiting the hypothesis that fungal-to-bacterial dominance characterizes turnover of soil organic matter and nutrients. Ecol. Monogr. 2015; 85: 457–472.

98. Zhou, J.; Jiang, X.; Zhou, B.; Zhao, B.; Ma, M.; Guan, D.; Li, J.; Chen, S.; Cao, F.; Shen, D. Thirty-four years of nitrogen fertilization decreases fungal diversity and alters fungal community composition in black soil in northeast China. Soil Biol. Biochem. 2016; 95: 135–143.

99. Farrer, E.C.; Suding, K.N. Teasing apart plant community responses to N enrichment: the roles of resource limitation, competition and soil microbes. Ecol. Letters 2016; 19: 1287-1296.

Figure captions

Fig. 1 Study site location.

Fig. 2 Pearson's correlations between soil properties and bacterial (A) and fungal (B) α-diversity.

Fig. 3 Relative abundances of dominant phyla (bacterial and fungal) in topsoil and subsoil. Notes: Control treatment in topsoil (CT_A), low nitrogen addition in topsoil (LN_A), high nitrogen addition in topsoil (HN_A); control treatment in subsoil (CT_B), low nitrogen addition in subsoil (LN_B), high nitrogen addition in subsoil (HN_B).

Fig. 4 Redundancy discriminate analysis (RDA) plots illustrating the relationships between the dominant bacteria (A) or fungal (B) phyla and soil physicochemical properties and microbial biomass.

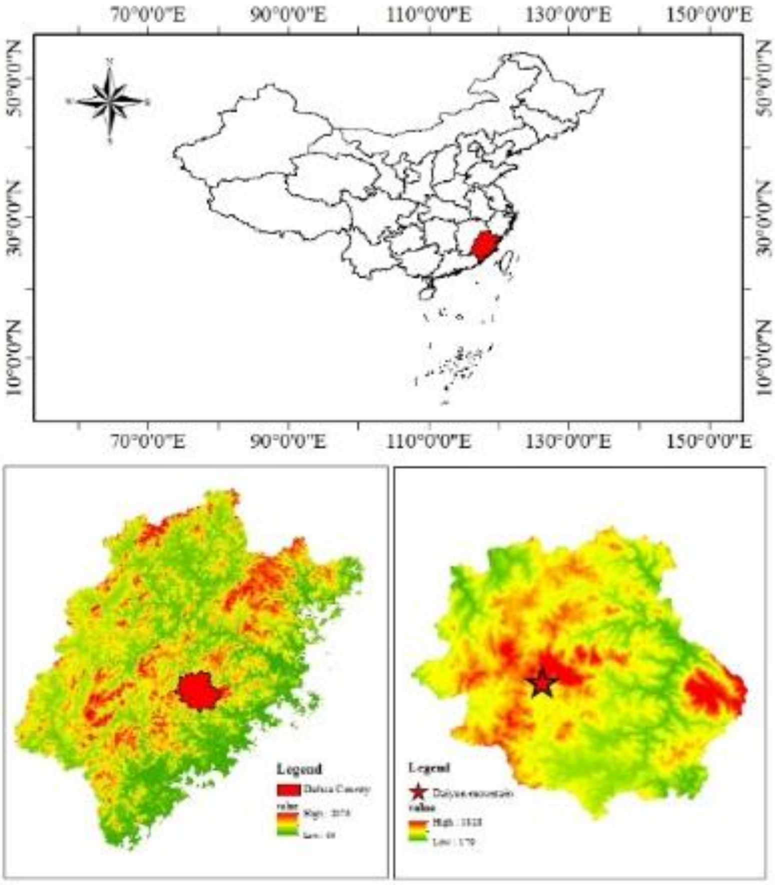
70°0'0"E
90°0'0"E
110°0'0"E
130°0'0"E
150°0'0"E
50°0'0"N
30°0'0"N
10°0'0"N
Legend
Legend

Fig. 1

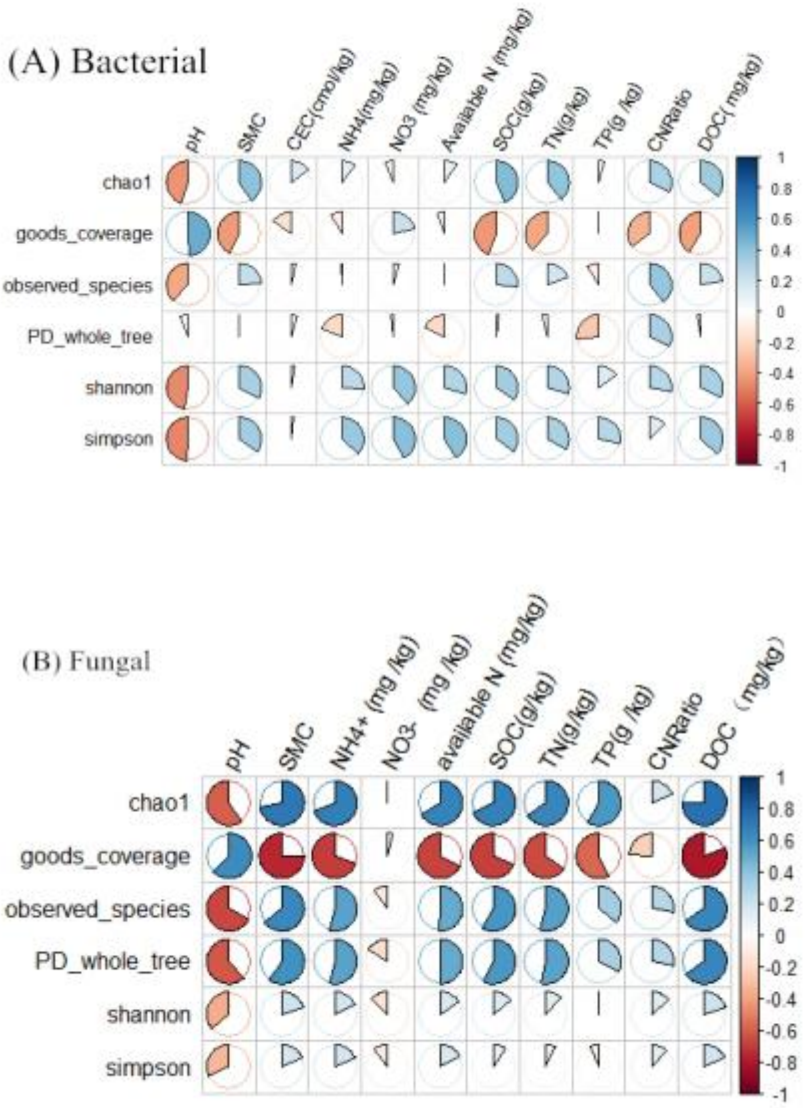
(A) Bacterial
pH
SMC
CEC(cmol/kg)
NH4(mg/kg)
NO3 (mg/kg)
Available N (mg/kg)
SOC(g/kg)
TN(g/kg)
TP(g /kg)
CNRatio
DOC(mg/kg)
chao1
goods_coverage
observed_species
PD_whole_tree
shannon
simpson
1
0.8
0.6
0.4
0.2
0
-0.2
-0.4
-0.6
-0.8
-1
(B) Fungal
pH
SMC
NH4+ (mg /kg)
NO3- (mg /kg)
available N (mg/kg)
SOC(g/kg)
TN(g/kg)
TP(g /kg)
CNRatio
DOC （mg/kg）
chao1
goods_coverage
observed_species
PD_whole_tree
shannon
simpson
1
0.8
0.6
0.4
0.2
0
-0.2
-0.4
-0.6
-0.8
-1

Fig. 2

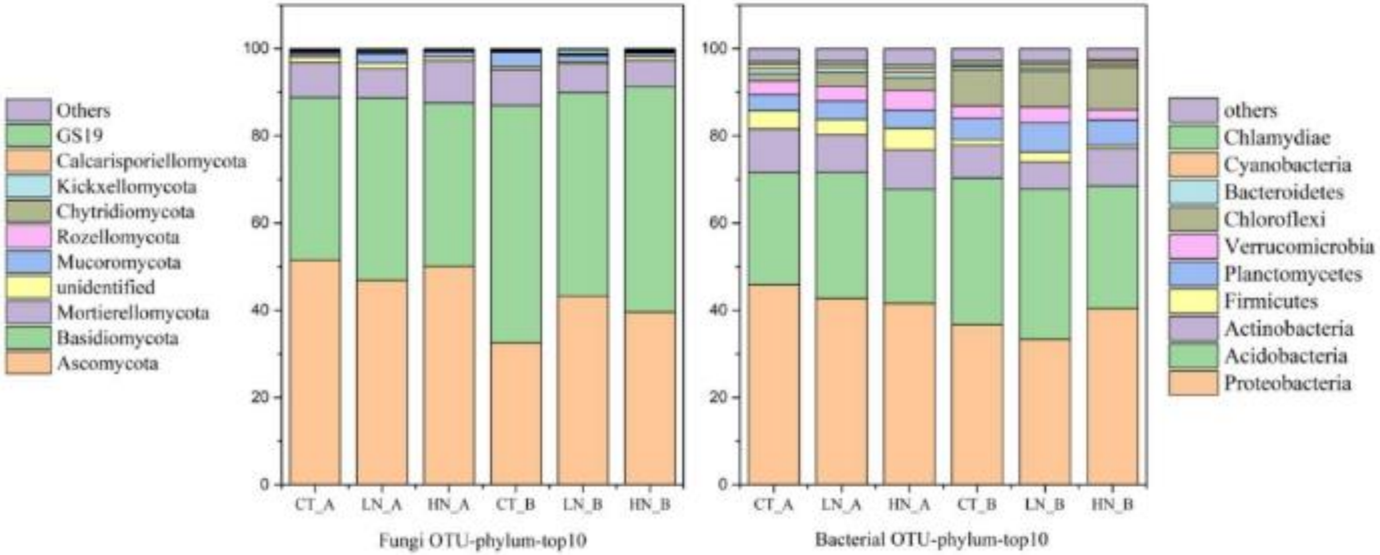
Others
GS19
Calcarisporiellomycota
Kickxellomycota
Chytridiomycota
Rozellomycota
Mucoromycota
unidentified
Mortierellomycota
Basidiomycota
Ascomycota
0
20
40
60
80
100
CT_A
LN_A
HN_A
CT_B
LN_B
HN_B
Fungi OTU-phylum-top10
0
20
40
60
80
100
CT_A
LN_A
HN_A
CT_B
LN_B
HN_B
Bacterial OTU-phylum-top10
others
Chlamydiae
Cyanobacteria
Bacteroidetes
Chloroflexi
Verrucomicrobia
Planctomycetes
Firmicutes
Actinobacteria
Acidobacteria
Proteobacteria

Fig. 3

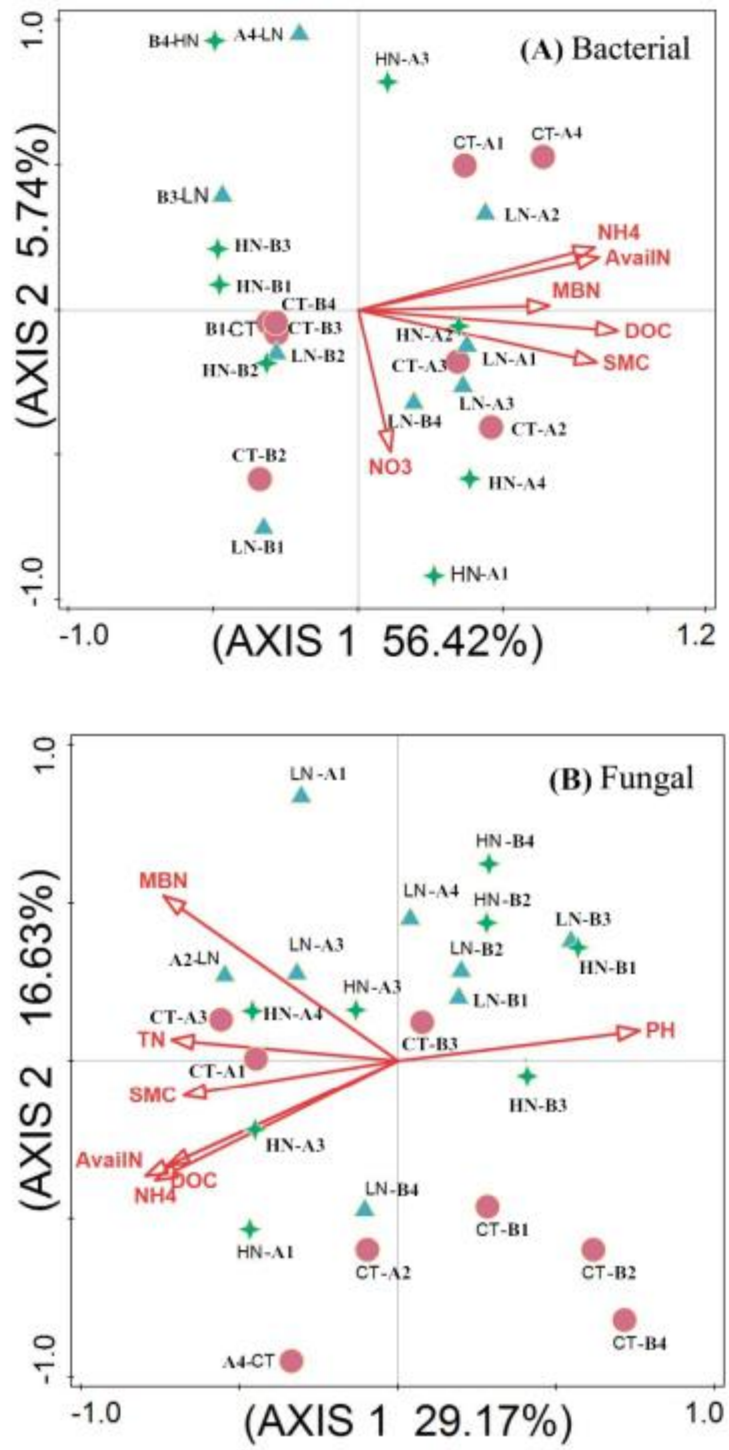
(A) Bacterial
(AXIS 2 5.74%)
(AXIS 1 56.42%)
1.0
-1.0
1.2
B4-HN
A4-LN
HN-A3
CT-A1
CT-A4
B3-LN
LN-A2
NH4
AvailN
HN-B3
HN-B1
MBN
CT-B4
B1-CT
CT-B3
HN-A2
DOC
LN-B2
LN-A1
HN-B2
CT-A3
SMC
LN-A3
LN-B4
CT-A2
NO3
CT-B2
HN-A4
LN-B1
HN-A1
(B) Fungal
(AXIS 2 16.63%)
(AXIS 1 29.17%)
LN-A1
HN-B4
MBN
LN-A4
HN-B2
LN-B3
LN-A3
LN-B2
HN-B1
A2-LN
HN-A3
LN-B1
CT-A3
HN-A4
PH
TN
CT-B3
CT-A1
SMC
HN-B3
HN-A3
AvailN
DOC
NH4
LN-B4
CT-B1
HN-A1
CT-A2
CT-B2
CT-B4
A4-CT

Fig. 4

Table 1 Responses of soil physicochemical properties at different soil depths to N addition in Daiyun mountain Nature Reserve, southeastern China.

	A horizon (topsoil)				B horizon (subsoil)			
	CT	LN	HN	p	CT	LN	HN	p
pH(H_2O)	4.22	4.32	4.35	0.43	4.57	4.60	4.58	0.80
pH(KCl)	3.85	3.91	3.87	0.75	3.71b	4.32a	4.05ab	0.10
SMC (%)	0.74	0.66	0.59	0.31	0.41	0.39	0.39	0.92
CEC (cmol kg^{-1})	12.58	9.47	10.63	0.49	12.27	8.61	8.79	0.09
NH_4^+ (mg kg^{-1})	74.28a	51.23b	62.77ab	0.06	30.32a	16.19b	29.61a	0.02
NO_3^- (mg kg^{-1})	4.80b	5.69ab	9.65a	0.07	6.22	5.49	6.45	0.47
Available N (mg kg^{-1})	81.68a	56.92b	72.42ab	0.03	36.53a	23.35b	36.05a	0.04
DOC (mg kg^{-1})	371.81	245.37ab	206.87b	0.11	63.92a	46.07ab	28.51b	0.11
SOC (g kg^{-1})	55.73	55.99	47.02	0.17	26.25	27.27	30.98	0.63
TN (g kg^{-1})	4.06	3.75	3.12	0.22	1.81	1.62	2.20	0.27
TP (g kg^{-1})	0.25	0.22	0.25	0.51	0.17	0.14	0.19	0.21
C:N	14.98	15.01	15.12	0.98	14.55	14.82	14.11	0.74
MBC	543.98	682.61	616.18	0.66	283.83	360.33	437.41	0.38
MBN	97.09c	201.99a	145.27b	0.00	50.31	61.78	81.91	0.15

The mean value from control (CT), low (LN), and high (HN) nitrogen addition treatments on A horizon (topsoil) and B horizon (subsoil) soil samples is shown. Different letters represent significant differences (one-way ANOVA, $p < 0.05$, LSD

post hoc analysis) between different levels of nitrogen addition.

Table 2 Effects of nitrogen deposition, soil layer, and their interactions on soil bacterial (B) and fungal (F) α diversity as indicated by two-way ANOVA statistics.

Diversity indices	Soil layer		Nitrogen treatme		Interaction	
	F	p	F	p	F	p
B_chao1	2.98	0.10	2.69	0.09	1.22	0.32
B_goods_coverage	3.17	0.09	4.60	0.02*	0.72	0.50
B_observed_species	2.02	0.17	1.66	0.22	3.54	0.05*
B_PD_whole_tree	0.001	0.97	1.57	0.24	5.80	0.01**
B_shannon	9.37	0.007**	0.70	0.51	5.67	0.01**
B_simpson	11.85	0.003**	0.37	0.70	3.51	0.05*
F_chao1	24.24	0.00***	1.39	0.27	1.65	0.22
F_goods_coverage	23.8	0.00***	3.58	0.04*	1.14	0.34
F_observed_species	12.85	0.002**	1.10	0.36	1.48	0.25
F_PD_whole_tree	13.14	0.002**	1.10	0.35	1.81	0.19
F_shannon	1.33	0.263	0.05	0.95	0.76	0.48
F_simpson	1.31	0.267	0.35	0.71	0.67	0.52

*****Correlation is significant at the 0.001 level.**

****Correlation is significant at the 0.01 level.**

*** Correlation is significant at the 0.05 level.**

Supplementary Materials

Fig. S1 The samples rarefaction curves of bacterial (A) and fungal (B) α-diversity. When the curves tends to be flat, the amount of sequencing data is reasonable, and more data will produce only a small number of new species.

Fig. S2 Partial Least Squares Discrimination Analysis（PLS-DA） of the soil bacterial (A,B) and fungal (C,D) community structure under different treatments in the topsoil and subsoil, which based on the abundance of OTUs used to determine the compositional variation. CT, LN, and HN, represent the control, low, and high nitrogen addition treatments.

Fig. S3 Bacterial (A) and fungal (B) species variation with different treatments analyzed by cladogram of Lefse analysis.

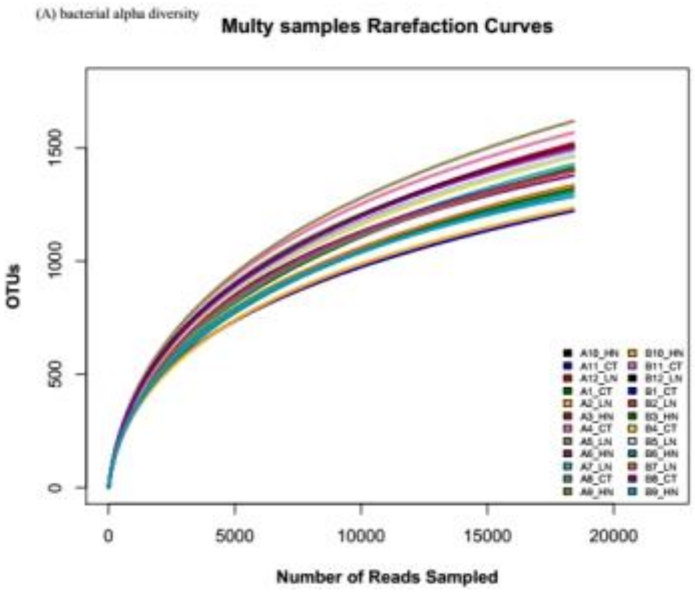
(A) bacterial alpha diversity
Multy samples Rarefaction Curves
OTUs
0
500
1000
1500
0
5000
10000
15000
20000
Number of Reads Sampled

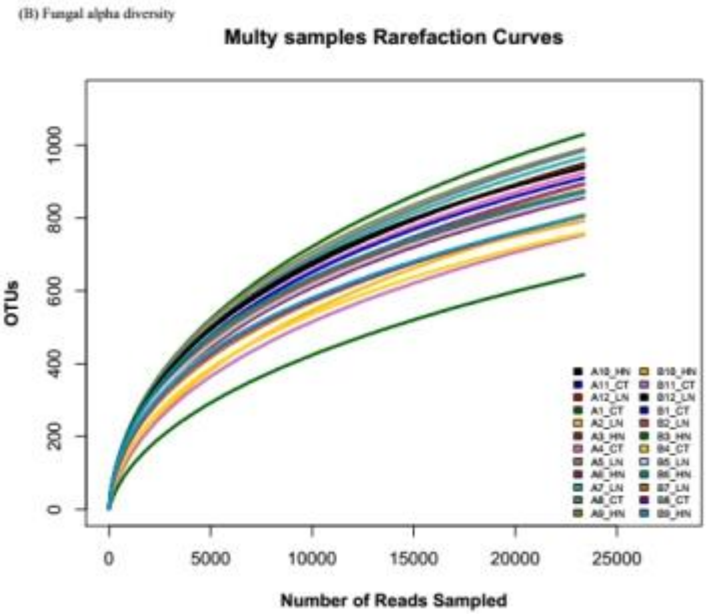
(B) Fungal alpha diversity
Multy samples Rarefaction Curves
OTUs
0
200
400
600
800
1000
0
5000
10000
15000
20000
25000
Number of Reads Sampled

Fig. S1

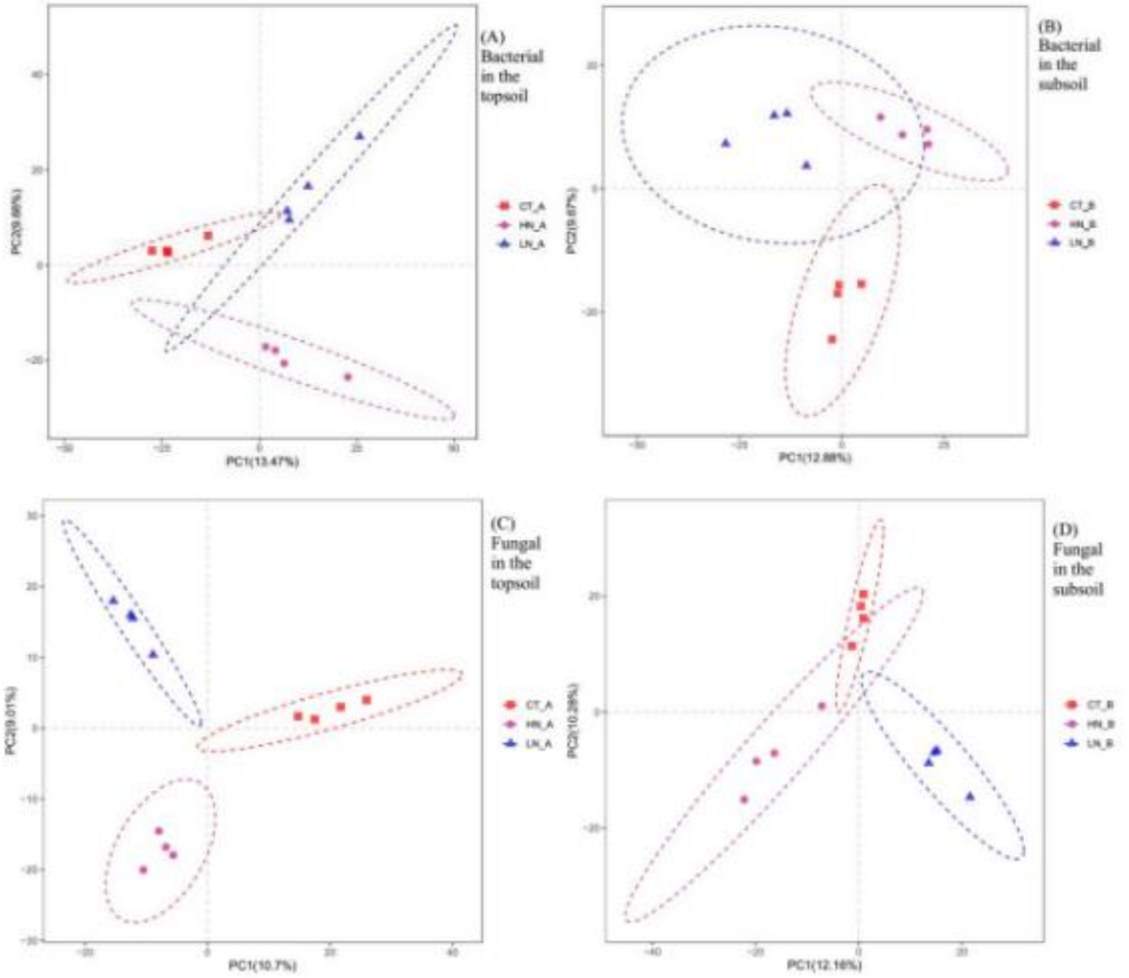
(A)
Bacterial
in the
topsoil
PC1(13.47%)
CT_A
HN_A
LN_A
(B)
Bacterial
in the
subsoil
PC1(12.88%)
CT_B
HN_B
LN_B
(C)
Fungal
in the
topsoil
PC1(10.7%)
CT_A
HN_A
LN_A
(D)
Fungal
in the
subsoil
PC1(12.16%)
CT_B
HN_B
LN_B

Fig. S2

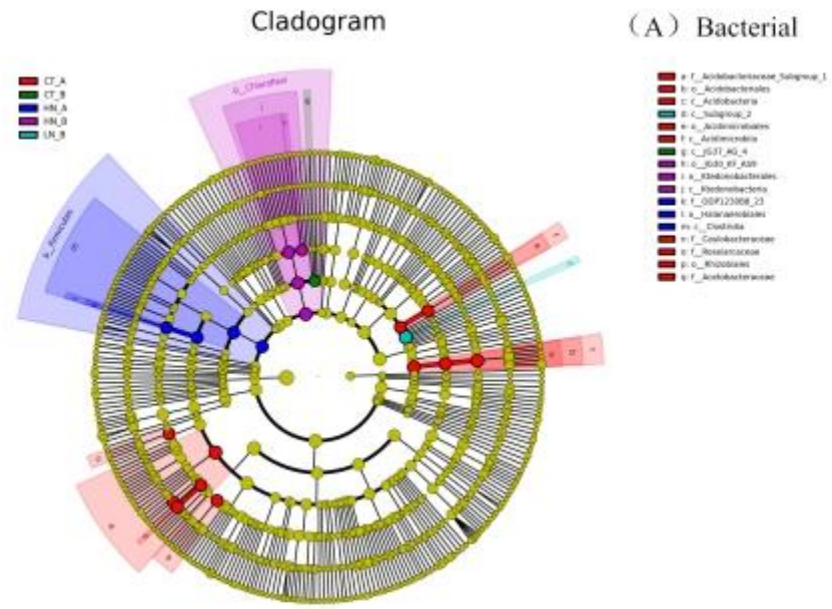
Cladogram
(A) Bacterial
CT_A
CT_B
HN_A
HN_B
LN_B

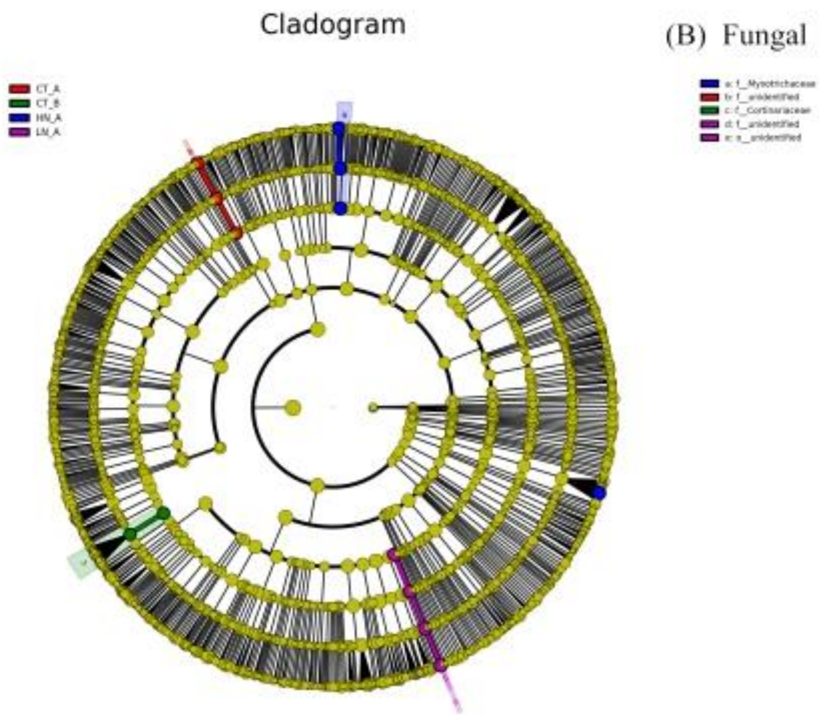
Cladogram
(B) Fungal
CT_A
CT_B
HN_A
LN_A

Fig. S3

Table S1 Primer sequences used in this study.

Primer Name	Primer sequence (5′-3′)
16S v3-v4 forward primer	GTACTCCTACGGGAGGCAGCA
16S v3-v4 reverse primer	GTGGACTACHVGGGTWTCTAAT
ITS1 forward primer	CTTGGTCATTTAGAGGAAGTAA
ITS1 reverse primer	TGCGTTCTTCATCGATGC

Table S2 Effects of different N-addition treatments at different soil depths based on 16S rDNA gene copy numbers, ITS gene copy numbers and fungi-to-bacteria ratios.

	16S	ITS	F:B
CT-A	1.20E+07	1.63E+06	0.250
LN-A	1.86E+08	3.80E+06	0.347
HN-A	1.39E+05	3.12E+05	0.801
CT-B	4.01E+08	3.41E+06	0.013
LN-B	1.05E+09	1.38E+06	0.002
HN-B	3.26E+07	2.87E+05	0.042

CT, control; LN, low nitrogen addition; HN, high nitrogen addition; A, topsoil; B, subsoil.

Table S3 Pearson's correlations of microbial abundance to soil properties and microbial biomass.

Soil properties	Bacterial abundance	Fungal abundance	Fungi-to-Bacteria ratio
pH（$H_2^{0)}$	0.385	0.164	-0.607**
pH (KCl)	0.776**	-0.037	-0.159
SMC	-0.38	0.231	0.440*
CEC	-0.305	0.466*	0.094
NH_4+^{-N}	-.493*	0.057	0.353
NO_3-^{-N}	-0.174	-0.29	0.508*
available_N	-0.459*	0.033	0.387
DOC	-0.32	0.101	0.391
SOC	-0.443*	0.163	0.498*
TN	-0.464*	0.222	0.458*
TP	-0.481*	0.133	0.333
CN Ratio	0.015	-0.179	0.3
MBC	-0.232	0.122	0.467*
MBN	-0.402	0.222	0.584**

Note: SMC, soil moisture content; CEC, soil cation exchange capacity; NH4+-N, ammonium N; NO3−-N, nitrate N; DOC, dissolved organic carbon; SOC, soil organic carbon; TN, total N; TP, total P.

*** *Correlation is significant at the 0.01 level**

*** Correlation is significant at the 0.05 level.**

Table S4 Good's coverage values of each group.

	Index	Bacterial goods_coverage	Fungal goods_coverage
Topsoil	CT	97.29%	98.43%
	LN	97.13%	98.50%
	HN	97.44%	98.56%
	p value	0.26	0.269
Subsoil	CT	97.30%	98.67%
	LN	97.40%	98.60%
	HN	97.60%	98.75%
	p value	0.03	0.066

CT, control; LN, low nitrogen addition; HN, high nitrogen addition

Table S5 Effects of N addition on soil bacterial and fungal α-diversity indices.

Diversity indices	Horizon A (Topsoil)				Horizon B (Subsoil)			
	CT	LN	HN	p	CT	LN	HN	p
B_chao1	1982.88	2100.99	1939.25	0.43	2010.63a	1925.25ab	1782.60b	0.05*
B_goods_coverage	0.97	0.97	0.97	0.26	0.97b	0.97b	0.98a	0.03*
B_observed_species	1399.70	1472.98	1458.68	0.64	1463.68a	1426.68a	1285.48b	0.00**
B_PD_whole_tree	98.94	104.14	104.37	0.42	106.97a	104.65a	96.04b	0.00**
B_shannon	8.32	8.30	8.46	0.33	8.31a	8.23a	7.99b	0.03*
B_simpson	0.99	0.99	0.99	0.17	0.99	0.99	0.99	0.22
F_chao1	1412.96	1373.87	1363.66	0.69	1192.84	1293.34	1165.80	0.12
F_goods_coverage	0.98	0.98	0.99	0.27	0.99ab	0.98b	0.99a	0.07
F_observed_species	958.00	909.43	906.23	0.49	807.23	874.68	778.45	0.24
F_PD_whole_tree	171.36	164.95	162.81	0.52	146.12	159.80	146.23	0.17
F_shannon	6.28	5.87	6.29	0.63	5.42	6.13	5.40	0.69
F_simpson	0.96	0.93	0.95	0.67	0.89	0.95	0.82	0.61

Table S6 Results of RDA interpretation of bacterial and fungal community by environmental factors.

**Bacterial community**	Explains %	Contribution %	pseudo-F	P
DOC	45.4	62.9	18.3	0.002
MBN	5.2	7.2	2.2	0.086
NO_3^-	3.5	4.8	1.5	0.168
Available N	3.1	4.2	1.4	0.224
SMC	4.1	5.7	1.9	0.102
NH_4^+	3.6	5.0	1.7	0.174
**Fungal community**	Explains %	Contribution %	pseudo-F	P
Available N	21.6	33.5	6	0.002
MBN	15.4	23.8	5.1	0.004
NH_4^+	5.3	8.3	1.9	0.1
pH	3.8	5.8	1.3	0.286
TN	2.7	4.2	1	0.412
DOC	2.2	3.5	0.8	0.58
SMC	3.1	4.9	1.1	0.364

DOC, dissolved organic carbon; MBN, microbial biomass N; SMC, soil moisture content; TN, total N.
