# Supplementary material for: Nutrient availability is a dominant predictor of soil bacterial and fungal community composition after nitrogen addition in subtropical acidic forests
Source: PLoS One. 2021 Feb 23;16(2):e0246263. doi: 10.1371/journal.pone.0246263 (PMC7901772; doi:10.1371/journal.pone.0246263)
Supplement: Attachment — Submitted filename: Respond to Reviewers.doc [file pone.0246263.s010.doc]

**Institute of Geography Sciences**

**Fujian Normal University, Fuzhou, China**

**yUEH m. chen**

**28-OCT-2020**

**Tel: +86-591-83483731**

**Fax: +86-591-83465397**

**E-mail:** [**ymchen@fjnu.edu.cn**](mailto:ymchen@fjnu.edu.cn)

**Julian Aherne**

**Academic Editor**

***Plos One***

Dear Editor:

We wish to submit a revised version of our manuscript, titled “**Nutrient availability is a dominant predictor of soil bacterial and fungal community composition after nitrogen addition in subtropical acidic forests**” [PONE-D-20-10592 (R1)], for publication in the *Plos One*. We have carefully revised the entire manuscript, especially the introduction and discussion, according to the reviewers’ comments, and have divided the discussion into subsections so that readers can better comprehend the main results. We have also reviewed the entire manuscript to improve the overall quality of the writing and have deposited sequencing data in NCBI Sequence Read Archive. The accession number for this data is PRJNA666625 and the SRP ID is SRP286064.

Our responses to the reviewers’ comments are presented below. The revised text in the re-submitted manuscript is written in red font.

Thank you for your consideration. I look forward to hearing from you.

Sincerely yours,

Yueh M. Chen

Professor of Geography Sciences

Fujian Normal University

Institute of Geography Sciences

**Response to Reviewer’s Comments**

**Editor’s Review**

**1. Please ensure that your manuscript meets PLOS ONE's style requirements, including those for file naming.**

**Response**: The manuscript has been revised with for consistency with Plos One guidelines for style. We have also provided figures and supplementary information in separate files according to guidelines and placed figure legends and tables at the appropriate places within the text. The writing has been substantially improved to meet your quality standards.

**2. Thank you for submitting the above manuscript to PLOS ONE. During our internal evaluation of the manuscript, we found significant text overlap between your submission and the following previously published works:**

https://peerj.com/articles/7631/

https://www.sciencedirect.com/science/article/abs/pii/S0048969717335684?via%3Dihub

**We would like to make you aware that** **copying extracts from previous publications, especially outside the methods section, word-for-word is unacceptable. In addition, the reproduction of text from published reports has implications for the copyright that may apply to the publications.**

**Please revise the manuscript to rephrase the duplicated text, cite your sources, and provide details as to how the current manuscript advances on previous work. Please note that further consideration is dependent on the submission of a manuscript that addresses these concerns about the overlap in text with published work.**

**We will carefully review your manuscript upon resubmission, so please ensure that your revision is thorough.**

**Response**: Thank you for your comments. We have rewritten the introduction and discussion and revised passages that may be similar to text from previous publications.

3. **In your Methods section, please provide additional information regarding the permits you obtained for the work. Please ensure you have included the full name of the authority that approved the field site access and, if no permits were required, a brief statement explaining why.**

**Response**: The corresponding author, Professor Yuemin Chen, has established a stable relationship with Daiyunshan Nature Reserve for cooperation in scientific research, and accordingly established an expert workstation within this reserve. Additionally, a member of the Administration Bureau of the Daiyunshan Nature Reserve is a co-author on this paper. Therefore, no permits were required for access to the field site. The sentence is on Page 9, Lines 145-146.

4. **In your Methods section, please provide additional location information, including geographic coordinates for the data set if available.**

**Response**: We have added accurate information about the longitude and latitude for the location on Page 8, Lines 125-127.

**5. We note that Figure 1 in your submission contain map images which may be copyrighted. All PLOS content is published under the Creative Commons Attribution License (CC BY 4.0), which means that the manuscript, images, and Supporting Information files will be freely available online, and any third party is permitted to access, download, copy, distribute, and use these materials in any way, even commercially, with proper attribution. For these reasons, we cannot publish previously copyrighted maps or satellite images created using proprietary data, such as Google software (Google Maps, Street View, and Earth). For more information, see our copyright guidelines: http://journals.plos.org/plosone/s/licenses-and-copyright.**

**Response**: Thank you for your comments. Figure 1 is drawn by Zeng Yaozhong using cartographic software (ArcGIS 10.2, Esri, USA). The map data is DEM elevation data downloaded from the geospatial data cloud of China.

Website: [http://www.gscloud.cn/home#page1/4](http://www.gscloud.cn/home#page1/4)

6. **We note that you are reporting an analysis of a microarray, next-generation sequencing, or deep sequencing data set. PLOS requires that authors comply with field-specific standards for preparation, recording, and deposition of data in repositories appropriate to their field. Please upload these data to a stable, public repository (such as ArrayExpress, Gene Expression Omnibus (GEO), DNA Data Bank of Japan (DDBJ), NCBI GenBank, NCBI Sequence Read Archive, or EMBL Nucleotide Sequence Database (ENA)). In your revised cover letter, please provide the relevant accession numbers that may be used to access these data. For a full list of recommended repositories,** see [http://journals.plos.org/plosone/s/data-availability#loc-omics](https://mail.126.com/js6/read/about:blank#loc-omics) or [http://journals.plos.org/plosone/s/data-availability#loc-sequencing](https://mail.126.com/js6/read/about:blank#loc-sequencing).

**Response**: Thank you for your comments. We have uploaded the data as requested in NCBI Sequence Read Archive. The accession number that can be used to access these SRA data is PRJNA666625 and the SRP ID is SRP286064.

7. **Please upload a new copy of Figure 1 as the detail is not clear. Please follow the link for more information:** <https://blogs.plos.org/plos/2019/06/looking-good-tips-for-creating-your-plos-figures-graphics/>

**Response**: Thank you for your comment. Figure 1 has been recreated to ensure that the details are clear.

**Response to Reviewer’s Comments	Reviewer # 1**

The authors would like to thank you for the valuable suggestions. The manuscript has been revised with due attention to your comments. The writing has been substantially improved to meet your publication quality standards.

**Specific Comments:**

**-The introduction of the manuscript is well written. But I found some recent published long-term studies on N fertilization in tropical/subtropical forests were not included. Particularly, a paragraph focusing on microbial community shift caused by N addition in subtropical forests could be added. I attach a few references and hope they can be incorporated into introduction/discussion.**

Wang et al., (2018) https://doi.org/10.1016/j.soilbio.2018.08.022
Wang et al., (2018) [https://doi.org/10.1016/j.soilbio.2018.03.009.](https://doi.org/10.1016/j.soilbio.2018.03.009. wangcong 13)
Wu et al., (2019) https://doi.org/10.1016/j.apsoil.2019.05.014
**Response**: We have revised the introduction according to your comments and have cited the suggested references [29-31]. We have also added a paragraph focusing on microbial community shift caused by N addition in subtropical forests. These revisions can be found on Page 5-6, Lines 74-90.

**-Methods did not describe measurement of CEC.**

**Response**: We have added details regarding the measurement of CEC in the Materials and Methods section. Please see the revised text on Page 11, lines 181-182.

**-For discussion of diversity, an explanation of why only bacteria diversity was affected in subsoil is needed. Particularly I found attributing diversity change to pH (L334) WAS NOT reasonable as I found it contradictory that HN treatment decreased bacteria α-diversity in subsoil significantly (Table S5), but subsoil pH did not show significant difference in CK and HN plots. Instead, subsoil pH was significant different between CK and LN plots (Table 1). And in L395 authors stated pH was not changed making it more confusing.**
**Response**: Thank you for the valuable comments. We have revised this paragraph as suggested. We have mentioned that DOC exerted the strongest effect on soil bacterial and fungal diversity, while pH does not appear to be as closely related to bacterial diversity. Please see the revised text on Page 25, Lines 419-420.

**-For discussion of community structure, does the community structure change somehow reflect observed functions? For instance, the observed decreased NH_4_ and increased NO_3_ in LN plots suggest potential high nitrification rate.**
**Response**: Thank you for the valuable comments. We have revised this statement as suggested and incorporated discussion regarding whether the changes in the nutrients may reflect differences in microbial community functions such as nitrification rate. Please see the revised text on Pages 28-29, Lines 478-499.

**-L428-431 I found the concluding remark is quite general, a stronger take home message is preferred. Also a lot of studies have studied biogeochemical cycling functions under N addition, it would make a more significant contribution by connecting functions with community structure.**

**Response**: We appreciate your suggestion. We have added a concluding paragraph giving a strong take-home message. Briefly, we mention that soil microbial community is affected by N addition, especially increasing oligotrophic bacteria and some microbial groups that play major roles in nutrient cycles. We have also highlighted the need for further studies connecting microbial community structure and functions and their interaction with biogeochemical cycles when subjected to N addition. Please see the revised text on Pages 29-30, Lines 508-524.

**-The writing needs substantial work. I encourage the authors to thoroughly check the manuscript to avoid grammatical mistakes and incomplete sentences. I also suggest authors remake some tables and figures. It is very hard to read figure legends in its current layout (e.g., Fig 1-3, Fig S1-3). Fig.2 does not have legend and caption does not provide enough information to interpret figure. Fig.4 does not have a legend and data labels have inconsistent font, also these labels make the figure messy. In Table 1, use superscript letters to avoid incomplete post-hoc results. Also I am very concerned about the reported soil moisture content (SMC<1%), authors should double check their data.
Response**: Thank you for the valuable comments. We have remade some tables and figures and added detailed figure legends and data labels. We have also attempted to make figure 4 less messy and maintain consistency in font. We have also used superscript letters for depicting post-hoc results in Table 1. Please see the revised figures 1-4 and tables 1-3. Regarding the soil moisture content, we inaccurately reported proportions instead of percentages and have now rectified this error.

**-L787-789 correlation?**
**Response**: We have revised this statement as suggested by the reviewer. Please see the revised text on Page 18, Lines 300 and refer to Tables 2 and S3 Table.

**-Please include all data (prior to statistical analysis) in supplement or deposit to a public repository for review.**

**Response**: We have uploaded the data as requested in the NCBI Sequence Read Archive. Accession number of this SRA data is PRJNA666625 and SRP ID is SRP286064.

**Response to Reviewer’s Comments	Reviewer # 2**

We thank the reviewer for the useful comments. We have extensively revised the manuscript according to your suggestions. We hope you will find the revised manuscript to be suitable for publication in Plos one.

**Abstract**
**Line 36, not exact.**

**Response**: Thank you for pointing this out. We have rewritten this sentence to clearly mention that soil dissolved organic C (DOC) and available N (AN) were significantly decreased after N additions in both topsoil and subsoil. Please see the revised text on Page 3, Lines 42-43.

**Line 40, add a sentence about the results' indication.**
**Response**: Thank you for your suggestion. We have added the sentence “This experiment provides further evidence in support of shifts in the diversity and structure of soil microbial communities in acidic subtropical forests in response to increasing nitrogen deposition.” Please see the revised text on Page 4, Lines 49-51.

**Introduction**
**The "Introduction" is lack of continuity in logic, and does not show how scientific questions are proposed. Some information should be given in " Discussion". Line 62-73, should be rewritten.
Response**: The authors would like to thank the reviewer for the valuable comments. The paragraph mentioned by the reviewer has been rewritten. We have added a paragraph focusing on microbial community shift caused by N addition in subtropical forests. Subsequently, we have mentioned the scientific questions and the accompanying hypotheses that we attempted to address. We hope that the revised version of the manuscript has better logical continuity.

**Material and methods**
**Line 107, subtropical. It is...**

**Response**: We have revised this statement as suggested. Please see the revised text on Page 8, Lines 128.

**Line 109, "Pinus taiwanensis" should be in italic.**

**Response**: Thank you for your advice. We have italicized all instances where this species is mentioned as suggested. Please see the revised text on Page 8, Lines 130-133.

**Line 211, the typeface is not consistent.**

**Response**: Thank you for identifying this inconsistency. We have ensured consistency in the font and spacing throughout the manuscript.

**Results**
**Line 218-228, the results should show the data rather than description.**

**Response**: Thank you for your comment. We have modified the results section accordingly to include data. Please see the revised text on Page 14, Lines 258-261.

**Line 223-224, the sentences should be rewritten. Is soil available N the sum of NH4+-N and NO3--N? Some values are the sum, and others are not in Table 1.**

**Response**: We have revised this statement as suggested. Available N is the sum of ammonium and nitrate and we have verified the values of these parameters. Please see the revised text on Page 10, 14 Lines 176-177, 262 and the new Table 1.

**Line 229, as a main result, it is not suitable that tables and figures in this section are not present in the main text. In fact, I think the results of soil bacterial and fungal gene copy numbers can be deleted in the text. Because the gene copy numbers are determined by the PCR cycles. The values are not in situ absolute values.**

**Response**: This paragraph has been deleted in the text according to the reviewer’s comment. We have now used copy numbers only to provide an estimate of relative abundance of different species. Please see the revised text on Page 13, Lines 262-263.

**Table 1, data should be shown as mean ± SE/SD.
Response**: We have revised the data as suggested. Please see the new Table 1.

**Discussion
Line 314-320, what indications of these previous studies for this study?**

**Response**: These previous studies indicated that there is no consensus on the response of soil microbial biomass to nitrogen addition in subtropical and tropical forest ecosystems. We have added such a statement in the discussion section. Please see the revised text on Page 24, Lines 399-400.

**Line 325-331, the "Discussion" should not repeat results simply.**

**Response**: This descriptive sentence has been deleted from the text and the possible implications of the results have been discussed instead. Please see the revised text on Pages 24-25, Lines 410-422.

**Response to Review Comments	Reviewer # 3**

The authors would like to thank you for the insightful comments. The manuscript has been substantially revised with careful consideration of your suggestions. I hope you will find the revised manuscript to be suitable for publication in Plos One.

1. **I can understand what the authors have written, but there are a lot of grammatic errors in this manuscript.**
   **Response**: The authors would like to thank you for the valuable comments. The manuscript has been rewritten and reviewed to ensure grammatical correctness.

**2. Although I like the three questions asked at the end of the Introduction, the section has not been well developed. I understand the structure of the section. Yet sentences within a paragraph were not tightly linked or well organized. Besides, the knowledge gap was not well developed and thus the significance of the work seems less appealing to me.**
**Response**: Thank you for your constructive comments. We have added a paragraph focusing on microbial community shifts caused by N addition in subtropical forests. Subsequently, we have presented our scientific questions and corresponding hypotheses. We hope that the organization of the text and the continuity of ideas have been improved in this version of the manuscript. I have added to the end of the introduction (beginning of last paragraph) the following sentence: “Although the effects of N addition have been studied, it is not clear if the changes in microbial community are mediated by direct increases in N availability, changes in the nutrient composition or acidification of the soil.” Please see the revised text on Page 5-6, Lines 74-90 and Page 7, Lines 105-108.

**3****. I encourage the authors to further explore the relationships among microbial communities and environmental parameters. Right now, they only used RDA to identify important variables for overall community composition. I cannot see the direction of the effects of these variables on specific microbial groups.**
**Response**: Thank you for your constructive comments. We have now used the Mantel test analysis to understand the association of the soil environmental factors with the composition of soil microbial communities. Please see the added test results on Page 22, Lines 379-384.

**4.The whole Discussion was terribly written. It was superficial and discrete most of the time. I really hope the authors could rewrite the whole section.**

**Response**: We have rewritten the entire discussion section as suggested by the reviewer. We hope there is greater depth and logical continuity in this revised version.

**Minor Revisions:**

1. **line27: You could clarify sampling time here.**

**Response**: We have revised this sentence to include the sampling time. Please see the revised text on Page 3, line 32.

2. **Line29: It would be more clear to specify how long had the N addition lasted before sampling for microbial analyses?**

**Response**: We have revised this statement as suggested. We sampled the soil sampling one year after N addition. Please see the revised text on Page 3, line 39.

3. **Line35: Spell the full word when it first appears.**

**Response**: We have ensured that all abbreviations have been expanded at first mention. We have clarified that DOC refers to soil dissolved organic carbon. Please see the revised text on Page 3, line 42-43.

4. **Line37-39: This result is too general. Clarify what variable is important for bacterial and fungal communities, respectively.**

**Response**: We found that DOC is most important for bacterial communities while AN is most important for fungal communities. We have modified the statement to include these details. Please see the revised text on Page 3, lines 45-47.

5. **Line50: Why is this negative?**

**Response**: Thank you for your question. Nitrogen addition has resulted in the reduction of understory vegetation. Therefore, this value is negative. This has been clarified. Please see the revised text on Page 5, lines 62.

6. **Line60: Why would you use additionally here? Do you have another reason explaining why N addition could increase MBC in subtropical forests?**

**Response**: We have removed the word “additionally” since it was not appropriate, as pointed out by the reviewer. Please see the revised text on Page 5, line 72.

7. **Line62: You should give this paragraph a topic sentence.**

**Response**: We have added the following sentence: N addition can also induce changes in soil microbial α-diversity and microbial community composition. Please see the revised text on Page 5, line 74.

8. **Line79: This sentence is not clear. Do you mean absolute abundance of major microbial groups were positively correlated with nutrient concentrations?**

**Response**: Yes, we have rewritten the sentence for clarity. Please see the revised text on Page 7, line 99-100.

9. **Line82: This sentence should go to the beginning of the paragraph.**

**Response**: We have moved this sentence as suggested. Please see the revised text on Page 6, line 91.

10. **Line89: I am quite confused by the first two sentences. What is your point here? Besides, many studies have investigated microbial responses to nutrient addition.**

**Response**: We have deleted these two sentences. Please see the revised text on Page 7.

11. **Line97-101: I like these questions. They are well linked.**

**Response**: Thank you for your appreciation.

12. **Line108: citation?**

**Response**: This sentence was quoted [45] from Chinese geography textbook. Author: Zhao Ji, Chen Chuankang. Press: Higher Education Press.

13. **Line119: Do these plots have similar elevations, topography/slope, and facing directions? I believe these factors all affect microbial communities. I know it may be not easy to find a big area with all the above-mentioned factors uniform on mountains. But the authors should introduce these conditions.**

**Response**: The experimental plots are close to the top of the mountain, face the same direction, and have similar slopes and elevations. We have mentioned this in the methods section. Please see the revised text on Page 9, Lines 147-148.

14. **Line135: Please clarify that you used air-dried soils**

**Response**: We have clarified this as suggested. Please see the revised text on Page 10, Lines 165.

15. **Line139:C should come first.**

**Response**: We have revised the statement as suggested. Please see the revised text on Page 10, Lines 168-169.

16. **Line143: Why would you use 'exchangeable' before ammonium and nitrate?**

**Response**: We have deleted the word “exchangeable.” Please see the revised text on Page 10, Lines 175.

17. **Line174: This paragraph is a little too simple compared the qPCR paragraph. I am not encouraging you to digging into every details, but at least we should see the key details here.**

**Response**: We have revised this paragraph as suggested by including relevant details. Please see the revised text on Page 12, Lines 209-213.

18. **Line175: Do you mean quality score per sequence? Clarify.**

**Response**: Yes, we did mean quality score per sequence. We have clarified this. Please see the revised text on Page 12, Lines 210-211.

19. **Line180: Did you rarefy the OTU table?**

**Response**: Yes, we have done so. The rarefaction curves are presented in S1 Fig.

20. **Line181: Version number?**

**Response**: The version number of this software is 10.3. We have included this detail as suggested. Please see the revised text on Page 12, Lines 219.

21. **Line184: Some repeated analyses in the first and third paragraphs are confusing me.**

**Response**: We have revised these paragraphs and removed redundant statements as suggested. Please see the revised text on Pages 12-13, Lines 222-229 and Page 13 Lines 239-243.

22. **Line192: Why PCA instead of more common ordination analyses in microbial ecology such as NMDS/RDA/CCA?**

#### Response: PCA is a method of ordination using only species composition, and is one of the unconstrained methods of ordination. CCA and RDA are two constricted ordination methods that use both species and environmental factors to ordinate data. We have provided an explanation for using this in the methods section on Page 13, Lines 231-232.

23. **Line193: Do you mean relative abundance of OTUs? If yes, clarify.**

**Response:** Here, we used the relative abundance of OTUs to calculate species composition and abundance of the microbes. We have provided the raw data in the S2 Table.

24. **Line219: Did you mention all these properties in the Materials and methods section? For example, total P and CEC.**

**Response**: We have added the methods that we used for estimating total P and CEC in the methods section. Please see the revised text on Page 10-11, Lines 170-182.

25. **Line224: Do you mean the sum of ammonium and nitrate? Please clarify.**

**Response**: Yes. Available N is the sum of ammonium and nitrate. Please see the revised text on Page 10, Lines 176.

26. **Line243: you said negative?**

**Response**: Yes. We have revised the sentence and added the minus sign. Please see the revised text on Page 15, Lines 271.

27. **Line247: It seems like you did not rarefy sequences before calculate alpha diversity indices. Can you justify this?**

**Response**: Thank you for your comment. The Allwegene sequencing company provided us with the following alpha diversity indices: Chao1 index, Observed species index, PD whole tree index, Shannon, Simpson index. The sequences were rarefied prior to calculating these indices. The total number of rarefaction is 18402. The rarefaction curves are presented in S1 Fig.

28. **Line268: What about the associations among fungal alpha diversity indices and soil properties? You showed the results in Fig. 2B but you totally neglected these results.**

**Response**: We have added sentences explaining these associations. The pH was negatively correlated with fungal Chao1, Observed species, and PD whole tree indices, and positively correlated with the good coverage index. Soil properties (SMC, NH_4_^+^, AN, SOC, TN, DOC) were negatively correlated with fungal diversity indices (Chao1, Observed species, PD whole tree) and positively correlated with the good coverage index. We also observed weak associations of Shannon and Simpson indices with soil properties. Please see the revised text on Page 19, Lines 308-314.

29. **Line776: Fig 4. Are these environmental parameters all significant? If not, please remove the insignificant ones.**

**Response**: Some of these parameters are statistically significant. We have removed the insignificant environmental parameters and re-drawn Figure 4. Please see the revised Figure 4.

**30. Line804: Fig. S3. By lefse, you mean LDA in the Materials and methods section? Please keep consistency throughout the manuscript.**

**Response**: Yes, we have clarified this method of analysis and consistently used LDA as suggested. Please see the revised text on Page 47, Lines 877-878.
