# Supplementary material for: Nutrient availability is a dominant predictor of soil bacterial and fungal community composition after nitrogen addition in subtropical acidic forests
Source: PLoS One. 2021 Feb 23;16(2):e0246263. doi: 10.1371/journal.pone.0246263 (PMC7901772; doi:10.1371/journal.pone.0246263)
Supplement: Attachment — Submitted filename: Respond to reviewers.docx [file pone.0246263.s011.docx]

**Response to Reviewer’s Comments	Reviewer # 1**

The authors would like to thank you for the valuable suggestions. The manuscript has been revised with due attention to your comments. The writing has been substantially improved to meet your publication quality standards.

**Minor Revisions:**

**-L28,31 change UNDERGROUND to BELOWGROUND**
**Response**: Thank you for the valuable comments. We have revised this statement as suggested. Please see the revised text on Page 3, Lines 29 and 32.

**-L205 MiSeq is used, but in results, authors used HiSeq, clarify.**

**Response**: Thank you for the comment. We have revised this statement as suggested. Please see the revised text on Page 17, Lines 286 and 288.

**-L353,430 What statistical analysis was used to compare community structure change under N treatments.**
**Response**: Thank you for the valuable question. One-way analysis of variance (ANOVA) was used to determine the differences in soil microbial relative abundance, and multiple comparisons were conducted using the least significant difference (LSD) test at a significance level of p < 0.05. Please see the revised text on Page 13, Lines 270–230.

**-L402-403 “ As soil microbes are mainly C limited [61], the increase of labile C input is expected to multiply microbial biomass.” The context was talking MBN, why authors bring up C? Labile C?**
**Response**: Thank you for the valuable question. C input led to the increase of MBC, but there was no statistical significance. Owing to CN coupling, MBN increases significantly. We have revised this statement as suggested. Please see the revised text on Page 24, Lines 411–413.

**-L477 use N loss to explain lack of response in fungi community does not make sense, it contradicts bacteria change.**

**Response**: Thank you for the valuable comments. This sentence cited the other authors’ views. We did not find the available evidence to support this view; hence, the sentence has been deleted in the text.

**-L499 this sentence lacks logic and evidence. Either elaborate it, or remove it.
Response**: Thank you for the valuable comments. The sentence has been deleted in the text as per the reviewer’s comment.

**-Fig 1. The study area should not have red shading, as the fig already has red color for elevation.**
**Response**: We have modified Fig 1 as suggested by the reviewer. Please see the revised text on Page 9, Line 142.

**Response to Reviewer’s Comments	Reviewer # 2**

We thank the reviewer for the useful comments. We have extensively revised the manuscript according to your suggestions. We hope you will find the revised manuscript to be suitable for publication in Plos One.

**Specific Comments:**

**1)** **"DOC and AN significantly decreased after N addition to both topsoil and subsoil" is not accurate.**

**Response**: Thank you for pointing this out. We have rewritten this sentence to clearly mention this and modified the result section. Please see the revised text on Page 3, 14, Lines 44–45, 265–267.

2) **Bacterial alpha-diversity in subsoil only decreased under high N addition treatment.**

**Response**: Thank you for your suggestion. We have made the necessary changes in the abstract and result sections. Please see the revised text on Page 3, 17, 30, Lines 46–47, 299–301, 515-517.

**3)** **Soil DOC is the most important environmental factor for bacterial community composition while AN is the most important factor for fungal communities" are not precise. Furthermore, the r value is very small (0.1-0.3).**

**Response**: The authors would like to thank the reviewer for the valuable comments. The paragraph mentioned by the reviewer has been rewritten. We have added MBN as the predictor in fungal community shift caused by N addition in subtropical forests. We hope that the revised version of the manuscript has better logical continuity. Please see the revised text on Page 3-4, 22, Lines 47–50, 385–393.

**Response to Review Comments	Reviewer # 3**

The authors would like to thank you for the positive comments. The manuscript has been slightly revised with careful consideration of other reviewers’ suggestions. I hope you will find the revised manuscript to be suitable for publication in Plos One.

**Specific Comments:**

**The authors put a lot of effort into improving the manuscript. Most of my concerns have been well addressed. I do not have further content-related comments.**

**Response**: The authors would like to thank you for the valuable and constructive comments.
